# An Assessment of Mobile Predator Populations along Shallow and Mesophotic Depth Gradients in the Hawaiian Archipelago

**DOI:** 10.1038/s41598-017-03568-1

**Published:** 2017-06-20

**Authors:** Jacob Asher, Ivor D. Williams, Euan S. Harvey

**Affiliations:** 1 Joint Institute for Marine and Atmospheric Research, University of Hawaii at Manoa, Honolulu, Hawaii United States of America; 2 Coral Reef Ecosystem Program, Ecosystem Sciences Division, Pacific Islands Fisheries Science Center, National Oceanic and Atmospheric Administration, Honolulu, Hawaii United States of America; 3 Curtin University, Department of Environment and Agriculture, Perth, Western Australia Australia

## Abstract

Large-bodied coral reef roving predators (sharks, jacks, snappers) are largely considered to be depleted around human population centers. In the Hawaiian Archipelago, supporting evidence is primarily derived from underwater visual censuses in shallow waters (≤30 m). However, while many roving predators are present or potentially more abundant in deeper strata (30–100 m+), distributional information remains sparse. To partially fill that knowledge gap, we conducted surveys in the remote Northwestern Hawaiian Islands (NWHI) and populated Main Hawaiian Islands (MHI) from 2012–2014 using baited remote underwater stereo-video. Surveys between 0–100 m found considerable roving predator community dissimilarities between regions, marked conspicuous changes in species abundances with increasing depth, and largely corroborated patterns documented during shallow water underwater visual censuses, with up to an order of magnitude more jacks and five times more sharks sampled in the NWHI compared to the MHI. Additionally, several species were significantly more abundant and larger in mesophotic versus shallow depths, which remains particularly suggestive of deep-water refugia effects in the MHI. Stereo-video extends the depth range of current roving predator surveys in a more robust manner than was previously available, and appears to be well-suited for large-scale roving predator work in the Hawaiian Archipelago.

## Introduction

Large-bodied, coral reef roving predators (e.g. sharks, jacks, and snappers) are generally believed to be depleted across much of their ranges, particularly close to human population centers^1–7^. Similarly, reduced numbers of sharks and large-bodied teleosts reflect comparable patterns in the heavily populated main Hawaiian Islands (MHI), with reef shark abundances estimated at 3–10% of natural baseline levels^7^ and populations of several jacks (e.g. *Caranx ignobilis* and *Caranx melampygus*) thought to be depleted as a result of fishing pressure over the past several decades^8–11^. This serves as a stark contrast to abundant roving predator groups found in the remote, difficult to access, and largely unpopulated (i.e. relatively lightly fished) Northwestern Hawaiian Islands (NWHI)^1, 12^.

The primary source of Hawaiian Archipelago large-bodied, shark, jack, and snapper abundance data comes from underwater visual censuses on open-circuit scuba in 30 meters or less^1, 12, 13^. However, these groups are also known to inhabit considerably deeper ‘mesophotic’ strata of 30–150 m or more, where information on predator movements and habitat use remains scarce^14–16^. For example, tiger sharks (*Galeocerdo cuvier*) and Galapagos sharks (*Carcharhinus galapagensis*) have been documented to depths greater than 200 m^17–21^, while whitetip reef sharks (*Triaenodon obesus*) have been recorded down to 330 m^22^. Other predators commonly observed during shallow water dive surveys in the NWHI, including the giant trevally (*Caranx ignobilis*) and the bluefin trevally (*Caranx melampygus*), have been found in waters to at least 188 m and 230 m respectively^9, 23, 24^. Mesophotic coral reefs (herein denoted as ‘MCEs’) and other mesophotic ecosystems ≥30 m may be partially shielded from environmental and anthropogenic influences impacting shallow water coral reefs between 0–30 m, and may serve as population reservoirs for predator species targeted by fishers in shallower depths^15, 16, 25, 26^. However, while mesophotic predator research has increased over the past two decades through the use of advanced sampling technologies, e.g. closed-circuit rebreather underwater visual surveys or acoustic/satellite tracking, predator assessments in Pacific mesophotic ecosystems remain largely unassessed in comparison with their shallower counterparts^15, 27–29^.

Given the documented evidence for higher roving predator abundance and/or biomass estimates in deeper waters around high-density human populations^25^, sparsely populated or remote areas^16, 30^, and the noted rarity or absence of several reef-associated shark species (e.g. sandbar sharks, *Carcharhinus plumbeus* and *Galeocerdo cuvier*) during diver surveys^31^, it’s feasible that open-circuit underwater visual censuses may be missing the bulk of their populations if surveys remain constrained to depths less than 30 m. Therefore, there is a clear need to extend research into deeper coral reef habitats in order to better understand patterns in distributions of roving predator in the Hawaiian Archipelago and elsewhere.

Baited remote underwater stereo-video systems (stereo-BRUVs; herein denoted as ‘BRUVS’) represent one alternative sampling tool to assess the relative abundance and size frequencies of roving predator populations. BRUVS can be deployed over a wide range of habitats and depth strata^32^, and can be used to generate highly accurate and precise length and abundance data for sharks, jacks, and other roving predators which are comparable to other survey methods^33–38^.

Here, we present results of roving predator BRUVS surveys across the Hawaiian Archipelago covering depths down to 100 m. Research objectives included the: (a) comparison of relative abundances and length-based distributions of major species contributing to roving predator assemblages across shallow and mesophotic depth strata in the MHI and NWHI; and (b) investigation of mesophotic habitats, which remain largely inaccessible to underwater visual censuses on open-circuit scuba, as possible ‘depth refugia’ (defined as areas protected from shallow water disturbances that may serve as potential reproductive population reservoirs) for MHI roving predator species considered rare in 0–30 m depths^26, 39^.

## Methods

### Ethics statement: Stereo-video sampling of reef fishes

This project was conducted under the Papahānaumokuākea Marine National Monument Research Permit no. PMNM-2013-018-M1, and meets the requirements of NOAA Administrative Order (NAO) Series 216-6, Environmental Review Procedures, Sections 5.05 and 6.03c.3 (a) for Categorical Exclusions (CE) for Research Programs (PIFSC-20120038) for survey activities in the Main and Northwestern Hawaiian Islands. Additional research clearance was granted by the National Environmental Policy Act (NEPA), Programmatic Environmental Assessment for Research Activities Conducted by the Coral Reef Ecosystems Program (CREP), Pacific Islands Fisheries Science Center (PIFSC), 2010–2015. Stereo-camera deployments conducted from 2012–2014 were completed under the University of Western Australia (UWA) animal ethics permit no. RA/3/100/1204 and the Curtin University animal ethics permit AEC-2014-09, in adherence to provisions contained within the Australian Code of Practice for the Care and Use of Animals for Scientific Purposes. All experimental stereo-video sampling protocols were approved in accordance with NOAA PIFSC CREP, UWA, and Curtin University research guidelines, permitting agencies, and/or animal ethics review committees as described above.

### Study Area

The Hawaiian Archipelago (Hawaii, USA), consisting of 18 islands and atolls stretching across a 2400 km SE-NW gradient, is one of the most isolated archipelagos in the world. The archipelago includes the Main Hawaiian Islands (MHI), which are geologically-young, high-islands subjected to heavy population and fishing pressures^1^, and the older, largely unpopulated NWHI composed primarily of sandy islets, atoll systems, and submerged shoals. In 2005, the State of Hawaii established the NWHI Marine Refuge, which closed all NWHI state waters to fishing. Protection was further enhanced by the establishment and subsequent expansion of the Papahānaumokuākea Marine National Monument (PMNM) in 2007 and 2016 respectively. Because of their management status and their remoteness, access is almost entirely limited to research and management groups and traditional Native Hawaiian practitioners.

### Survey Operations and Site Selection

**Figure 1:**
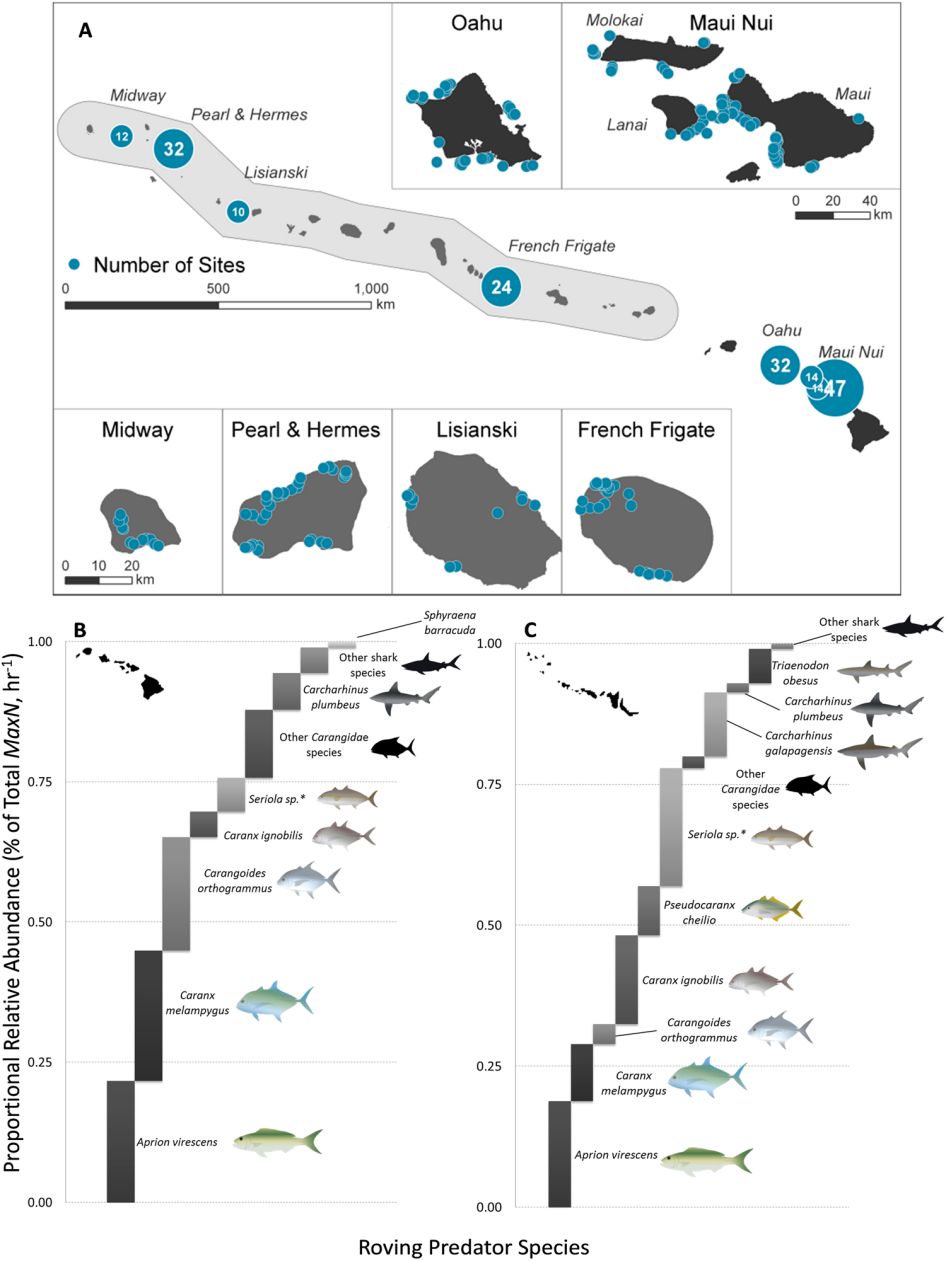
(**A**) Map indicating BRUVS sampling locations across the Hawaiian Archipelago. Map generated in ArcGIS, 10.3 (http://www.esri.com/software/arcgis) by Tomoko Acoba (NOAA PIFSC CREP). (**B**) Proportional relative abundance (% of total *MaxN*, hr^−1^) of total predator assemblages in the MHI and (**C**) NWHI. *Indicates numerical abundance of *Seriola dumerili*, *Seriola rivoliana*, *and Seriola sp*. (excluding *Seriola lalandi*) pooled. *Staggered bar plots* generated in MS Excel 2010 (https://products.office.com/en-us/microsoft-excel-2010). Maps in the figure were generated using Adobe Illustrator CS5 (https://www.adobe.com/products/illustrator.html).

Four of the MHI (Oahu, Maui, Molokai, Lanai) were sampled during two NOAA research expeditions in September and October 2012, with additional Oahu shore-based small boat sampling efforts completed in November 2013. Subsequent deployments in the NWHI (French Frigate Shoals, Lisianski, Pearl and Hermes Reef, Midway Atoll) were conducted during two NOAA research expeditions in May and September 2014 (Fig. 1). During each sampling effort, sites were selected in ‘mesophotic’ (30–100 m) and ‘shallow water’ (0–30 m) forereef and fringing reef habitats. Shallow water sites were randomly selected from locations previously surveyed by SCUBA divers conducting routine monitoring operations for reef fish and roving predators^40, 41^, with there being at least an hour between the completion of diver surveys and deployment of baited camera stations. Mesophotic survey sites were randomly selected from a pool of 500 × 500 m grid cells generated from bathymetric and backscatter data products produced by the University of Hawaii, School of Earth and Ocean Sciences (SOEST), Hawaii Mapping Research Group (Main Hawaiian Islands Multibeam Bathymetry and Backscatter Synthesis, http://www.soest.hawaii.edu/HMRG/multibeam/). Grid cells were constrained within a 100 m contour line using data derivatives from SOEST HMRG 50 m bathymetry and topography grid cells, and stratified into three predetermined, near “equally spaced” depth bins (30–53 m, 53–76 m, 76–100 m). Because the primary goal was to compare among hard-bottom habitats, grid cells containing backscatter values with >35% unconsolidated sediment (sand; obtained from SOEST HMRG 60 m backscatter grid cells) were excluded from the site pool. However, at some locations (esp. the MHI), bottom type information was not available or was inaccurate, leading to sampling of unconsolidated sediment (sand flats).

All BRUVS surveys were completed between 0800–1600, with soak times of 60 minutes, and all sampling sites separated by at least 500 meters.

### Stereo-video collection and data processing

The BRUVS used in this study followed the design of Harvey *et al*.^42–44^, and were constructed from a pair of high definition Sony handheld video cameras with a wide-angle lens adaptor, held in waterproof housing and mounted on a base bar 0.7 m apart, inwardly converged at 8°. Prior to and following each research mission, each BRUVS was calibrated using CAL^TM^ software according to protocols described elsewhere^43–46^. The oily fish Japanese sanma (*Cololabis saira*) was used as bait, which was pulped and loaded into 800 g wire-mesh baskets attached 1.2 m from the stereo-cameras prior to deployments.

Upon completion of BRUVS deployments, all video footage was converted from MT2S to AVI format using the program Xilisoft^TM^, followed by the annotation of stereo-video imagery with EventMeasure-Stereo^TM^ videographic software^45^. Species were identified to their lowest possible taxonomic level, with relative abundance recorded as *MaxN* measures. *MaxN*, defined as “the maximum number of fish belonging to each species present in the field of view of the cameras at one time”^47–52^ is a conservative abundance measure that avoids repeated counts of the same targets. Length-based measurements were derived by making nose-to-tail fork length measurements (FL) in EventMeasure at the time of *MaxN*. To ensure the accuracy and precision of measurements, and for consistency with established BRUVS protocols, *MaxN* and length measurements were limited to targets within 7 m of the stereo-cameras^53^. All *MaxN* and fork-length data were compiled and cataloged according to the National Fisheries Information System (FIS) Information Portal practices^54^.

Deployments were excluded from analysis when the field of view was ≥ 30% obstructed – i.e. if BRUVS had flipped and were facing straight down or straight up, if they were blocked by upright substrate, or when visibility dropped below 7 m, which occurred for a number of MHI sites in <6 m depth. A subtotal of 107 baited sites in the MHI and 78 sites in the NWHI were sampled (185 pooled deployments; Fig. 1, top panel). Outputs from efforts by region and depth strata are listed in Table 1.

**Table 1:** Summary of sampling effort in the Hawaiian Archipelago, detailing the number of sites per region, depth strata, and habitat type.

Location	Depth (m)	Hard-bottom	Soft-bottom	Total Sites
Main Hawaiian Islands	0–30	38	2	40
	30–53	24	5	29
	53–100	10	28	38
	Subtotal	72	35	107
Northwestern Hawaiian Islands	0–30	27	—	27
	30–53	19	3	22
	53–100	23	6	29
	Subtotal	69	9	78
	**Total surveys**	**141**	**44**	**185**

### Target groups

Analysis of BRUVS surveys was focused on high-level roving predators, with selections based on assignments as described in Friedlander and DeMartini^1^, Holzwarth *et al*.^13^, Parrish *et al*.^30^, and Williams *et al*.^12^. These included all shark species, large-bodied non-planktivorous jack species (Carangidae), the great barracuda (*Sphyraena barracuda*), and the green jobfish (*Aprion virescens*).

### Environmental variables

Depth data was obtained from UWATEC dive gauges attached to the stereo-camera base bar. Habitat type was visually classified based on video footage into one of 9 categories: aggregate reef, spur and groove, pavement, rock/boulder, reef rubble, aggregate patch reef, sand with scattered coral/rock, or sand flat (100% unconsolidated sediment)^24^. Habitat complexity was visually estimated on a five-point scale: 1 = flat, no vertical relief; 2 = low and sparse relief; 3 = low but widespread relief; 4 = moderately complex; and 5 = very complex with numerous fissures and caves^55^. Finally, percent cover of hard coral, soft coral, macroalgae, turf algae, crustose coralline algae, and sand was visually-estimated from video imagery using the NOAA PIFSC CREP fish team benthic classification protocol^56^.

## Data Analysis

### Experimental Design

Roving predator abundance and fork length-based measurements were examined according to two *a priori* factors: *Region* (MHI and NWHI: two levels, fixed) and *Depth strata* (shallow water (0–30 m); upper mesophotic (30–53 m); lower mesophotic (53–100 m); three levels, fixed). The decision to combine 53–76 m and 76–100 m abundance estimates *post*-*hoc* into a single level (lower mesophotic) came as a result of the reduced number of MHI hard-bottom mesophotic sites encountered below 53 m, with upper/lower mesophotic depth stratification aligning with coral reef fish assemblage structures observed in the MHI (Asher, unpublished data) and reported elsewhere^39, 57^. A third *post*-*hoc*, two-level fixed factor was added (*Habitat*; hard-bottom, unconsolidated sediment), as coral reef roving predators were commonly sighted in both substrate types which precluded the exclusion of BRUVS surveys that sampled sand flats. Finally, length data was pooled into two comparative depth strata (0–30 m; 30–100 m) because of small sample sizes.

### Statistical analyses

Multivariate roving predator assemblage analysis was conducted on a zero-adjusted Bray-Curtis (B-C)^58^ dissimilarity matrix using square root transformed relative abundance data using PRIMER v7.0.11 with the PERMANOVA+ add on software^59, 60^. Segregated regional and depth-inferred differences between roving predator population aggregates were first obtained through the bootstrapping function^61, 62^, and visualized as a metric multidimensional scaling (mMDS)^63^ ordination with bootstrap regions set to 95% confidence intervals (plotted as ellipses), a Kruskal stress formula set to 1, and minimum stress assigned to 0.01. A successive Hierarchical Cluster Analysis^64^ was calculated from distanced-dissimilarities between group centroids (region x depth x habitat) in order to visualize potential effect sizes and their interactions, with the original dissimilarities (distances between individual centroids) compared against cophenetic dissimilarity (distance between centroid clusters). Akin to a suitability index, a cophenetic correlation of r > 0.8 can be interpreted as a strong representation of the original centroid dataset^65^.

Changes to MHI and NWHI roving predator assemblages were evaluated along the continuous depth gradient within each respective region using a canonical analysis of principal coordinates (CAP). Subsequent CAPs were used to examine the efficacy of *a priori* MHI and NWHI depth group assignments through “leave-one-out” cross validation and allocation of observations to groups^66, 67^. Finally, Person’s rank correlations of individual species recording >0.35 were superimposed (as vectors) with the resultant CAP axes within each respective region as additional exploratory measures^59^.

Variation in assemblage structure between regions, depth strata, and habitat types were further tested using a Permutational Multivariate Analysis of Variance (PERMANOVA) as this is a robust test for examining correlations within potential heterogeneous variances^68^. A random, mixed three-way design PERMANOVA with 9999 permutations, constructed using Type III sum of squares (SS) was carried out. If factor effects or their interactions were significant, additional PERMANOVA pair-wise comparisons were conducted to investigate levels of significance within and between factor levels, with Monte Carlo p-values used for cases with fewer than 30 unique permutations^59^. Because PERMANOVA can remain sensitive to differences in multivariate dispersions, tests for dispersion homogeneity within groups (permutation of dispersions, PERMDISP), with 9999 permutations, were conducted in concert with PERMANOVA to further assess the variability of sampling regions against different depth and habitat strata.

A shade plot/heat map^69^ was constructed to further delineate abundance distributions of individual species across regions and depth strata, with sites ordered along the x-axis according to region and increasing depth. The y-axis was constructed according to roving predator groups, which were first standardized, transformed into a distance-based resemblance matrix using Whittaker’s Index of Association, and plotted via Hierarchical Cluster analysis^64^ using group average clustering and a Type III similarity profile (SIMPROF) analysis^70^ with 9999 permutations. A Similarity Percentages, Species Contributions (SIMPER) test^71, 72^ was then used to identify the predominant species similarities/dissimilarities within and between regional and depth strata factors, along with the percentage of species which explained similarities/dissimilarities.

For species that provided significant contributions to those identified in the SIMPER tests, additional univariate PERMDISP and PERMANOVAs using Euclidean distance measures were conducted on square root transformed relative abundance data. For univariate non-parametric analyses of *Seriola* species, *Seriola dumerili* and *Seriola rivoliana* abundance totals were pooled together (pooling herein denoted as “*Seriola sp* ^†^”) along with individuals marked “*Seriola sp”* that couldn’t be differentiated between the two. *Seriola rivoliana*, which had distinctly different characteristics than other members of the *Seriola* genus, were rarely encountered and were excluded from pooling.

Finally, changes to the relative abundance of individual species identified in SIMPER were modeled along continuous depth gradients using R statistical software (version 3.3.0) following the approach used by Fukunaga *et al*.^73^, generating non-parametric quantile regression splines through the *rq* () function in the *quantreg* package^74, 75^.

### Length-based estimates

Differences in length distributions for those species identified in SIMPER output were compared between respective regions (MHI, NWHI) and depth strata (shallow [0–30 m], mesophotic [pooled 30–100 m strata]) using untransformed raw length data (no zeros) across model factors and the non-parametric Kolmogorov-Smirnov test^76^, with data pooled from all mesophotic depths due to insufficient target species length measurements in upper or mesophotic strata alone. Average fork-length estimates (mm) were obtained for several species, including *Aprion virescens*, *Caranx melampygus*, *Caranx ignobilis*, *Carcharhinus galapagensis*, and *Triaenodon obesus*. All other species were measured, but excluded from analysis due to insufficient fork-length sampling pools.

### Habitat Characterization

Environmental relationships between regions and depth strata were visualized through bootstrapping from the original sampling pool. Bootstraps were plotted as a metric multidimensional scaling (mMDS) ordination, with bootstrap regions set to 95% confidence interval ellipses, a Kruskal stress formula set to 1, and minimum stress assigned to 0.01. In order to further gauge the ecological relationships between shallow water and upper and lower mesophotic zones, a Principal Component Analysis (PCA)^77, 78^ was performed on normalized environmental variables separately for the MHI and NWHI.

Finally, linkages between normalized, Euclidean-distance based environmental matrices and roving predator assemblage (abundance) matrices in the MHI and NWHI were explored using distance-based linear modeling (DISTLM) in PERMANOVA+^59, 79^, with the most parsimonious model constructed using modified Akaike’s Information Criterion (AICc) and *BEST* procedures. DISTLM allows for the testing of variation within predator assemblages to be explained through multiple environmental predictor variables, generating the most parsimonious models from the lowest AIC values. Distance-based redundancy analyses (dbRDA) were then used to construct constrained ordinations from *BEST* fitted values from the MHI and NWHI, using linear combinations of environmental variables which best explained the variation within roving predator assemblages belonging to each respective region^59^.

## Results

### Roving Predator Assemblage Description

**Figure 2:**
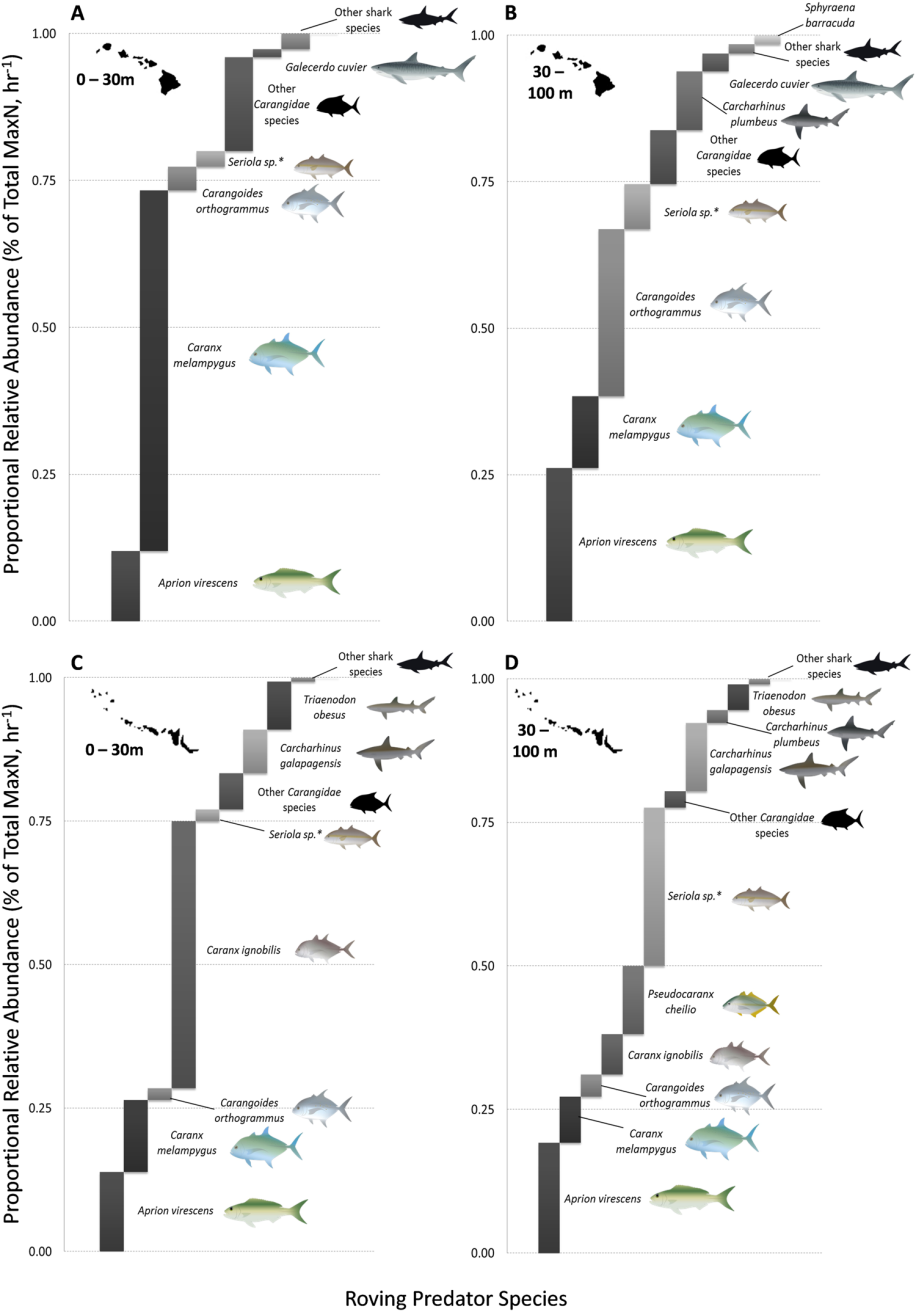
(**A**) Shallow and (**B**) Mesophotic pproportional relative abundance (% of total *MaxN*, hr^−1^) of predator assemblages in the MHI. (**C**) Shallow and (**B**) Mesophotic pproportional relative abundance of predator assemblages in the NWHI. *Staggered bar plots* generated in MS Excel 2010 (https://products.office.com/en-us/microsoft-excel-2010). Maps in the figure were generated using Adobe Illustrator CS5 (https://www.adobe.com/products/illustrator.html).

A total of 198 individual roving predators were recorded over 107 BRUVS samples in the MHI (mean and SE: 1.85 ± 0.27), while 425 roving predators were recorded over 78 BRUVS sites in the NWHI (5.45 ± 0.84, Table 2). The snapper *Aprion virescens* was the most common roving predator species overall, comprising a large proportion of the pooled roving predator community in each region (22% MHI, 19% NWHI; Fig. 1, lower panel). However, as a collective group, Carangidae comprised 65% of all roving predators belonging to ten species in the MHI (1.22 ± 0.19 SE, Table 2), with *Caranx melampygus* dominating shallow water abundances (51%, Fig. 2A) and *Carangoides orthogrammus* remaining prevalent in mesophotic depths (27%, Fig. 2B). Similarly, eight species of Carangidae accounted for 61% of all observations in the NWHI (3.33 ± 0.70 SE), with *Caranx ignobilis* dominating shallow waters (40%, Fig. 2C), and *Seriola sp* ^†^ comprising the major group (28%) in mesophotic habitats (Fig. 2D). Finally, sharks formed 12% and 20% of MHI and NWHI roving predator abundances respectively (Table 2, Fig. 2). In total, 22 sharks belonging to 4 species were recorded in the MHI (0.21 ± 0.05 SE), with sandbar sharks (*Carcharhinus plumbeus*) encompassing the majority of all shark sightings (59%) and another 23% of sightings belonging to tiger sharks (*Galeocerdo cuvier*). In contrast, 85 sharks belonging to 5 species were recorded in the NWHI (1.09 ± 0.14 SE), with Galapagos (*Carcharhinus galapagensis*; 56%) and whitetip reef sharks (*Triaenodon obesus*; 30%) comprising the majority of encounters. Neither species were sampled by BRUVS in the MHI.

**Table 2:** Average abundance (mean *MaxN*), standard error (SE), and NWHI:MHI abundance ratios for roving predator species sampled in the Hawaiian Archipelago.

Species	Common Name	Main Hawaiian Islands (MHI)						Northwestern Hawaiian Islands (NWHI)						Abundance Ratio
		n	Mean *MaxN*	% Drops	Min. depth (m)	Max.depth (m)	Islands	n	Mean *MaxN*	% Drops	Min. depth (m)	Max.depth (m)	Islands	NWHI:MHI
**Barracuda** (**Sphyraenidae**)														
*Sphyraena barracuda*	Great barracuda	2	0.02 ± 0.01	1.9	30.2	53.9	Molokai	—	—	—	—	—	—	—
**Snappers** (**Lutjanidae**)														
*Aprion virescens*	Green jobfish	43	0.41 ± 0.11	26.2	13.4	94.8	All	80	1.03 ± 0.16	73.1	2.7	100.0	All	2.51
**Jacks** (**Carangidae**)														
Main species														
*Carangoides orthogrammus*	Island jack	40	0.37 ± 0.13	15.9	17.1	96.6	Maui, Lanai, Oahu	15	0.19 ± 0.08	9.0	5.5	50.3	FFS, LIS, PHR	0.51
*Caranx melampygus*	Bluefin trevally	46	0.43 ± 0.09	26.2	3.0	46.6	All	43	0.55 ± 0.14	34.6	2.7	50.3	All	1.28
*Caranx ignobilis*	Giant trevally	9	0.08 ± 0.03	7.5	30.8	80.8	Maui, Molokai, Oahu	67	0.86 ± 0.29	28.2	2.7	50.3	All	10.75
*Pseudocaranx cheilio*	Thick-lipped jack	—	—	—	—	—	—	37	0.47 ± 0.31	10.3	40.5	100.0	PHR, MID	—
*Seriola dumerili*	Greater amberjack	6	0.06 ± 0.03	4.7	42.7	92.0	Molokai, Oahu	55	0.71 ± 0.39	14.1	23.5	93.3	FFS, LIS, PHR	11.83
*Seriola rivoliana*	Almaco jack	6	0.06 ± 0.03	2.8	21.3	92.0	Molokai, Oahu	27	0.35 ± 0.17	16.7	24.7	100.0	LIS, PHR, MID	5.83
Unidentified *Seriola sp*.*		—	—	—	—	—	—	7	0.09 ± 0.04	7.7	60.4	—	FFS, LIS, PHR	—
Subtotal *Seriola sp*.**		12	0.12 ± 0.05	6.5	21.3	92.0		89	1.13 ± 0.42	29.5	23.5	100.0		9.42
Other species														
*Alectis ciliaris*	Threadfin jack	5	0.05 ± 0.02	0.9	58.5	—	Oahu	—	—	—	—	—	—	—
*Carangoides ferdau*	Barred jack	4	0.04 ± 0.03	1.9	14.3	30.5	Maui, Oahu	1	0.01 ± 0.01	1.3	37.8	—	FFS	0.25
*Elagatis bipinnulata*	Rainbow runner	1	0.01 ± 0.01	0.9	14.9	—	Lanai	—	—	—	—	—	—	—
*Gnathanodon speciosus*	Yellow trevally	2	0.02 ± 0.01	1.9	14.3	42.4	Maui, Oahu	—	—	—	—	—	`	—
*Scomberoides lysan*	Queenfish	3	0.03 ± 0.02	2.8	3.05	30.5	Lanai, Maui, Oahu	—	—	—	—	—	—	—
*Seriola lalandi*	Yellowtail amberjack	—	—	—	—	—	—	5	0.06 ± 0.03	2.6	65.5	85.3	LIS, PHR	—
Unidentified Carangidae		9	0.08 ± 0.04	4.7	4.6	96.6	Maui, Oahu	3	0.04 ± 0.03	2.6	43.6	55.8	FFS, PHR	0.50
Subtotal *Other species*		24	0.22 ± 0.05	11.2	4.6	96.6		9	0.12 ± 0.06	6.4	37.8	85.3		0.55
Subtotal all jacks		131	1.22 ± 0.19	48.6	4.6	96.6		260	3.33 ± 0.7	70.5	2.7	100.0		2.73
**Sharks** (**Carcharhinidae**)														
Main species														
*Carcharhinus galapagensis*	Galapagos shark	—	—	—	—	—	—	48	0.62 ± 0.15	30.8	6.1	81.1	All	—
*Carcharhinus plumbeus*	Sandbar shark	13	0.12 ± 0.05	8.4	54.9	95.1	Maui, Molokai, Oahu	7	0.09 ± 0.03	9.0	55.8	93.3	FFS, LIS, PHR	0.75
*Triaenodon obesus*	Whitetip reef shark	—	—	—	—	—	—	26	0.14 ± 0.05	25.6	5.8	61.6	All	—
Other species														
*Galeocerdo cuvier*	Tiger shark	5	0.05 ± 0.02	4.7	4.6	55.8	Maui, Molokai, Oahu	1	0.01 ± 0.01	1.3	86.6	—	PHR	0.20
*Carcharhinus amblyrhynchos*	Grey reef shark	2	0.02 ± 0.01	1.9	24.1	68.3	Maui, Oahu	1	0.01 ± 0.01	1.3	73.2	—	FFS	0.50
*Carcharhinus melanopterus*	Blacktip reef shark	1	0.01 ± 0.01	0.9	14.9	—	Lanai	—	—	—	—	—	—	—
Unidentified shark		1	0.01 ± 0.01	0.9	57.0	—	Oahu	2	0.03 ± 0.02	2.6	38.1	73.2	FFS, LIS	3.00
Subtotal *Other species*		9	0.08 ± 0.03	7.5	4.6	68.3		4	0.05 ± 0.03	3.8	38.1	73.2		0.63
Subtotal all sharks		22	0.21 ± 0.05	15.9	4.6	95.1		85	1.09 ± 0.17	55.1	5.8	93.3		5.19
**Total mobile predators**		198	1.85 ± 0.27	67.3				425	5.45 ± 0.84	85.1				2.95

% Drops indicate the percentage number of BRUVS deployments where a species or group is observed. FFS: French Frigate shoals, LIS: Lisianski, PHR: Pearl and Hermes Reef, MID: Midway. *Seriola sp. that could not be differentiated between *Seriola dumerili* and *Seriola rivoliana*. ^†^pooled totals of *Seriola dumerili*, *Seriola rivoliana*, and unidentified *Seriola sp*.

**Figure 3:**
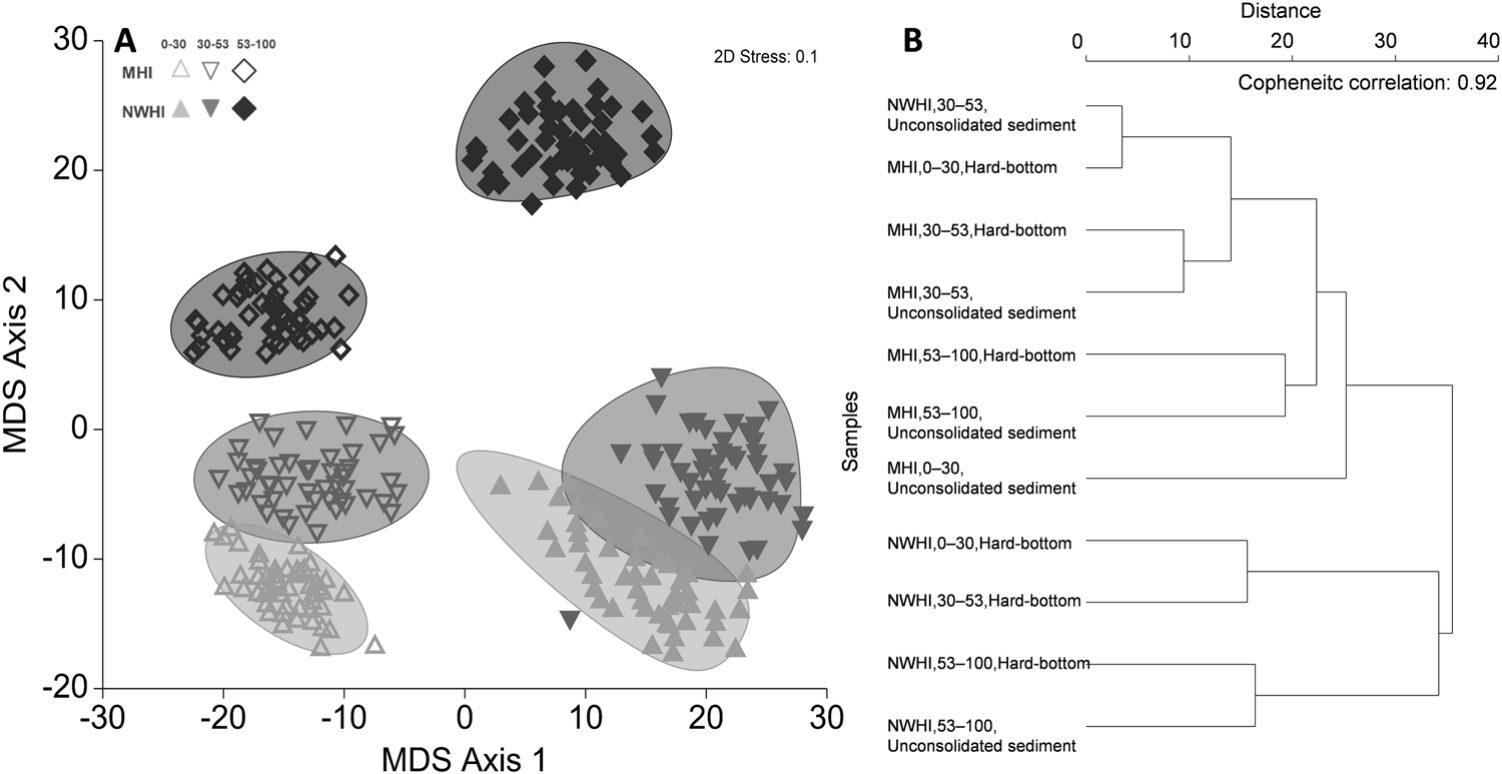
(**A**) *Bootstrap resampling plot*, 50 bootstraps per group. Square root transformed, zero-adjusted Bray Curtis roving predator abundance data (*MaxN*, hr^−1^) by Region (MHI, NWHI) x Depth Strata (SPC; upper and lower mesophotic), plotted metric multi-dimensional scaling (mMDS). Shaded bootstrap regions, which represent measurements of centroid error: 95% confidence ellipses, averages based on m = 10 dimensional metric MDS (rho = 0.985). Open symbols represent MHI sites, closed symbols represent NWHI sites. Light grey = shallow water (0–30 m), medium grey = upper mesophotic (30–53 m), dark grey = lower mesophotic (53–100 m). (**B**) Hierarchical cluster analysis dendrogram of group centroids f by Region (MHI, NWHI), Depth (0–30 m, 30–53 m, 53–100 m), and Habitat (Hard-bottom, unconsolidated sediment). Note the absence of NWHI unconsolidated sampling sites between 0–30 m. Cophenetic correlation = 0.92.

Roving predator assemblages differed between regions and depth strata (Global PERMANOVA, both p = 0.0001, Table 3). The relationship between roving predator assemblage structures and continuous depth gradients among the 107 surveys in the MHI (δ² = 0.35, m = 4 principal coordinate axes, Supplementary Materials, Figure S1A) and 78 surveys in the NWHI (δ² = 0.55, m = 3 principal coordinate axes, Figure S1B) confirmed a high degree of community overlaps between depths, particularly between 0–30 and 30–53 m (Fig. 3A, Supplementary Materials, Table S2). When examining the efficacy and cross validation of depth-zone assignments within the MHI and NWHI, 63% and 97% of assignments in the lower mesophotic zone were correctly made in the MHI and NWHI respectively, with misclassification errors largely driven by assignment switches (i.e. assemblage overlaps) between shallow water and upper mesophotic groups (Supplementary Table S1, Figure S1C,D), with MHI patterns being particularly susceptible to leave-one-out allocation errors due to the greater number of zero sightings or singleton predator observations during BRUVS surveys.

**Table 3:** PERMANOVA tests of pooled roving predator abundance (all species), *Aprion virescens*, *Caranx melampygus*, and *Carangoides orthogrammus* between region (Re), depth (De), and habitat strata (Ha).

All Roving Predators (Pooled)					*Aprion virescens*			*Caranx melampygus*			*Carangoides orthogrammus*		
Source	df	MS	Pseudo-F	P(perm)	MS	Pseudo-F	P(perm)	MS	Pseudo-F	P(perm)	MS	Pseudo-F	P(perm)
Re	1	8792.3	7.4288	**0**.**0001**	3.8974	11.803	**0**.**0004**	0.02812	0.098861	0.7538	0.3993	1.5871	0.2125
De	2	4637.8	3.9186	**0**.**0001**	0.16104	0.4877	0.5934	2.5871	9.0951	**0**.**0013**	1.3098	5.206	**0**.**0053**
Ha	1	1944.4	1.6429	0.1496	0.004282	0.012968	0.905	0.52007	1.8284	0.1765	—	—	—
RexDe	2	2298.2	1.9418	**0**.**0333**	0.15957	0.48325	0.6142	0.24641	0.8663	0.4208	0.35809	1.4233	0.2437
RexHa	1	1288.8	1.089	0.3755	0.026249	0.079494	0.7679	0.1228	0.43171	0.5026	—	—	—
DexHa	2	1740.6	1.4707	0.1481	0.56074	1.6982	0.1685	0.38141	1.3409	0.2476	—	—	—
RexDexHa**	1	3202.1	2.7056	**0**.**0189**	0.86488	2.6192	0.0996	0.1228	0.43171	0.4995	—	—	—
Res	174	1183.5			0.3302			0.28445			0.2516		
***Caranx ignobilis***				***Carcharhinus plumbeus***				***Seriola sp***.†					
Source	df	MS	Pseudo-F	P(perm)	MS	Pseudo-F	P(perm)	MS	Pseudo-F	P(perm)			
Re	1	5.7601	20.21	**0**.**0001**	0.005267	0.060992	0.8088	3863.6	19.597	**0**.**0001**			
De	2	1.9644	6.8924	**0**.**0017**	1.3877	16.071	**0**.**0001**	2751.8	13.958	**0**.**0001**			
RexDe	2	3.8234	13.415	**0**.**0001**	0.005578	0.064598	0.9381	1247	6.3253	**0**.**0014**			
Res	179	0.28501			0.086348			197.15					
***Pseudocaranx cheilio***					***Carcharhins galapagensis***			***Triaenodon obesus***					
Source	df	MS	Pseudo-F	P(perm)	MS	Pseudo-F	P(perm)	MS	Pseudo-F	P(perm)			
De	2	1.3364	3.2205	**0**.**0468**	0.3151	0.71497	0.4901	1.4012	5.7816	**0**.**0046**			
Ha	1	0.25834	0.62255	0.3612	0.43735	0.99237	0.3353	—	—	—			
DexHa**	1	0.55416	1.3354	0.194	1.4101	3.1995	0.0755	—	—	—			
Res	73	0.41497			0.44072			0.24235	(Note Res df = 66)				

PERMANOVA tests of *Caranx ignobilis*, *Carcharhinus plumbeus*, and Seriola sp** are presented for region x depth strata, following preliminary three-factor tests, and for *Carcharhinus galapagensis* and *Triaenodon obesus* between depth and habitat strata in the NWHI. Figures in bold indicate significant results. Total number of permutations per cell exceed 9700 except for the univariate factor test (depth) for *Triaenodon obesus*.

Upon assessing the unbalanced sampling of hard-bottom vs. unconsolidated sediment sites, assemblage patterns between group centroids largely mirrored as previously described (Fig. 3B) with outliers attributed to small sample sizes for those strata (MHI unconsolidated sediment: n = 2, 0–30 m and NWHI: n = 3, 30–53 m). Interactive effects were disproportionately driven by intra- and inter-regional differences highlighted in successive pair-wise tests (Supplementary Material, Table S2).

**Figure 4:**
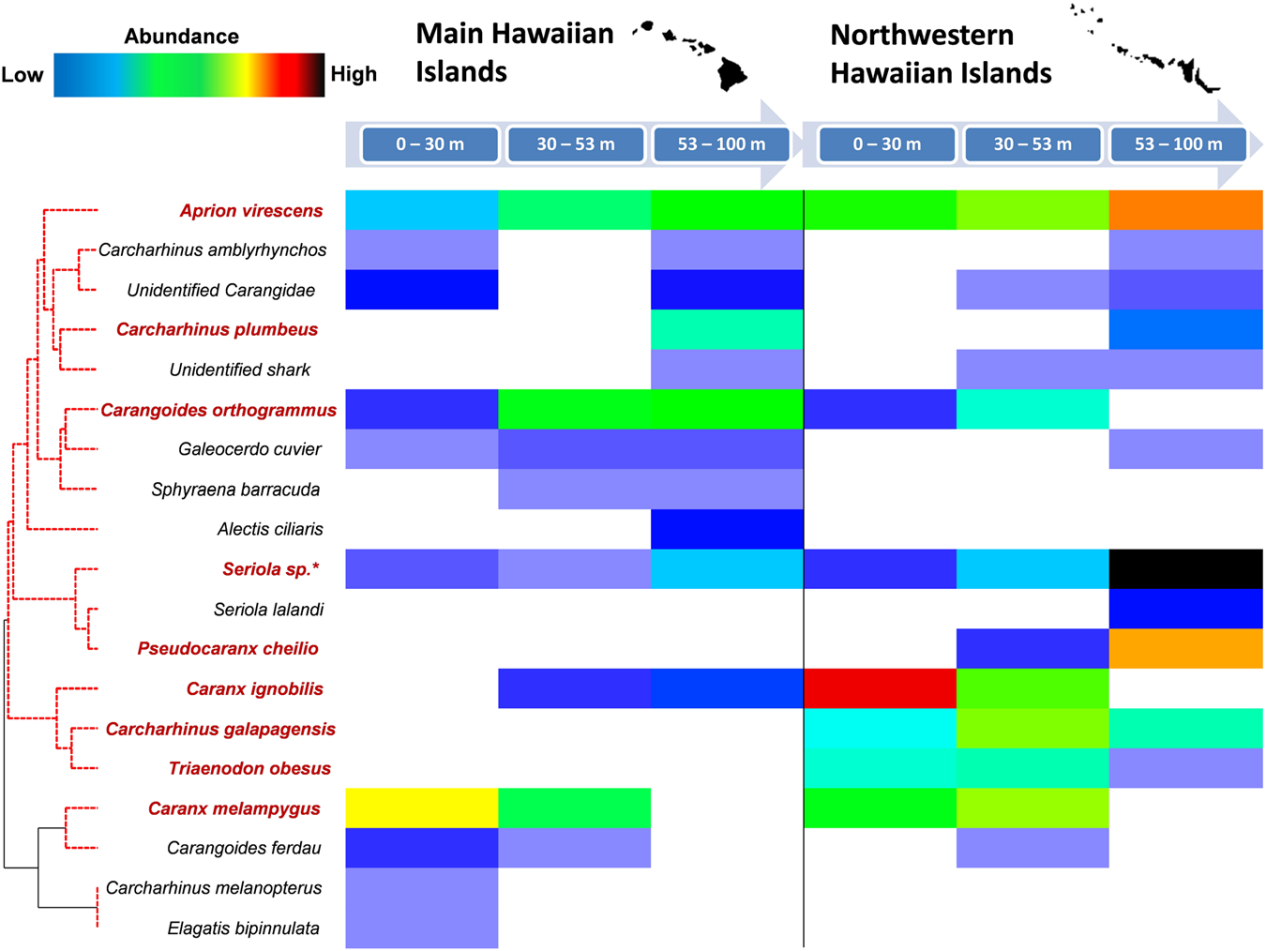
*Shade plot* showing regional (MHI, NWHI) and depth distributions of all roving predator species. Raw species relative abundance values (*MaxN*, hr^−1^: color ramped blocks) were square root transformed to down-weight more abundant species. Y-axis (roving predator resemblance): position of standardized *MaxN*, hr^−1^ predator values, ranked by Whittaker’s Index of Association transformation and Group Average Hierarchical Cluster analysis (Type III SIMPROF, with permutation between sites). Red dotted lines: groups of coherent species. Species identified in SIMPER highlighted in red. (b) X-axis: Sites grouped according to region (MHI, NWHI) and depth strata, aligned from left to right. Shade plot generated in PRIMER 7.0.11 (http://www.primer-e.com/). Maps in the figure were generated using Adobe Illustrator CS5 (https://www.adobe.com/products/illustrator.html).

Finally, the prominent species identified in SIMPER similarity/dissimilarity measures and shade plot outputs (Fig. 4) largely drove assemblage differences between regions and depth strata. These included *Aprion virescens*, *Caranx melampygus*, *Carangoides orthogrammus*, *Caranx ignobilis*, *Triaenodon obesus*, *Carcharhinus galapagensis*, *Pseudocaranx cheilio*, *Seriola sp* ^†^., and *Carcharhinus plumbeus*. Comparable with MHI CAP outputs, *Caranx melampygus* appeared aligned with shallow and upper mesophotic sites, while *Carangoides orthogrammus* and *Aprion virescens* were encountered in higher abundances in upper and lower mesophotic zones (Fig. 4, Supplementary Material, Figure S1C, lower panel). *Caranx melampygus* presented a similar pattern in the NWHI, with *Carnagoides orthogrammus*, *Caranx ignobilis*, *and Triaenodon obesus* likewise remaining more prevalent in shallow and upper mesophotic zones. In contrast, *Aprion virescens* and *Carcharhinus galapagensis* remained more broadly distributed between depth strata, although greater abundances were noted for both species in mesophotic depths. Finally, *Seriola sp*.^†^, *Pseudocaranx cheilio*, *and Carcharhinus plumbeus* remained prevalent in the upper and/or lower mesophotic zones, being near-absent from shallow-water strata (Fig. 4, Supplementary Material, Figure S1D, lower panel).

### Roving Predator Abundances: Univariate analysis

**Figure 5:**
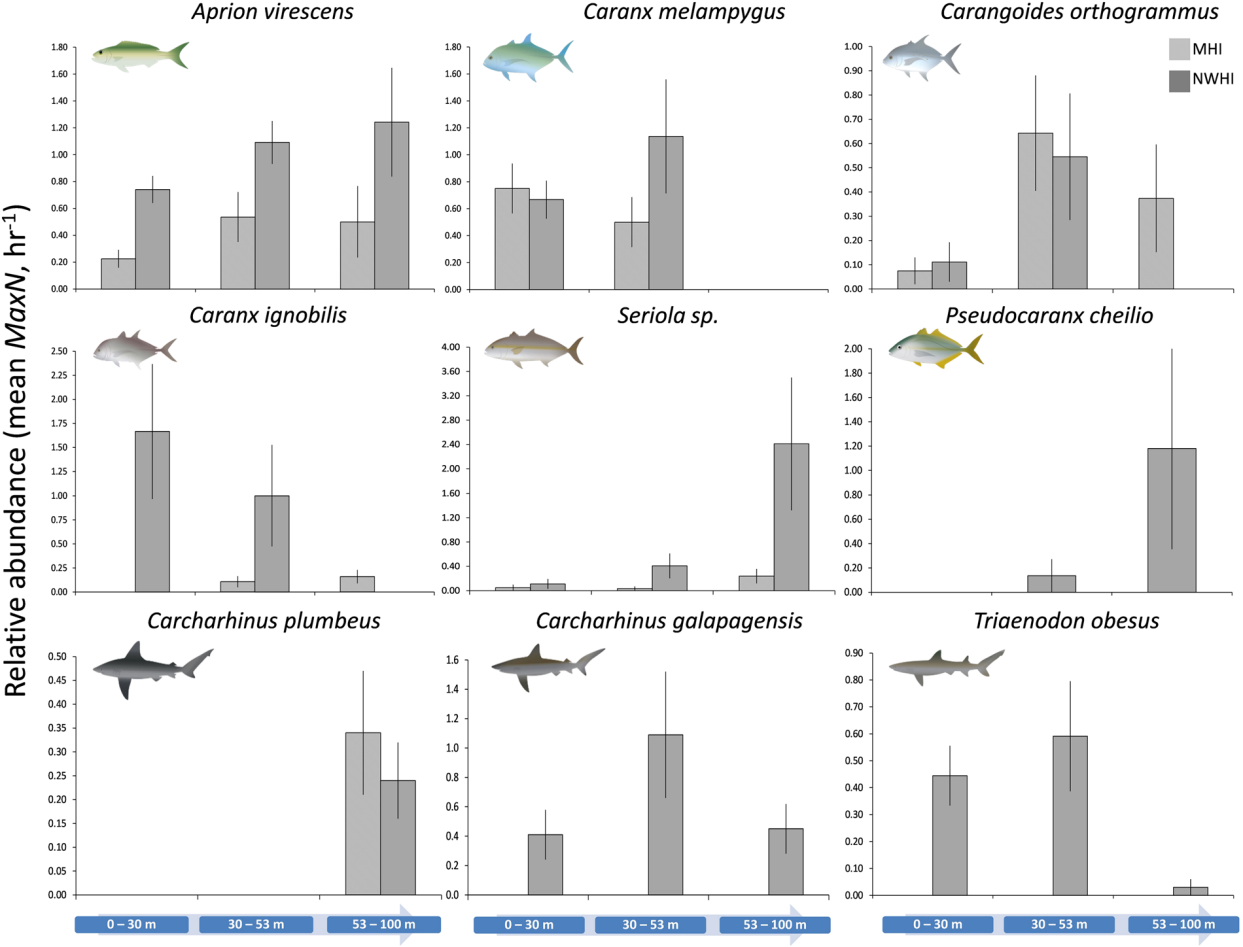
Mean relative abundance (Mean *MaxN*, hr^−1^ ± SE) of roving predator species identified in SIMPER analyses across regions and depth strata (habitats pooled). Depth is ordered in increasing intervals, with all habitats pooled. Light grey = MHI, dark grey = NWHI. Note the differences in scales along the y-axis.

*Aprion virescens* were homogeneously dispersed (p > 0.05) across all depth and habitat strata (Tables 3–4; Fig. 5, top left), recording significant regional differences (p < 0.001; 2.5 times greater abundance in the NWHI versus MHI) irrespective of depth strata or inclusion/exclusion of habitat as a pooled covariate. In contrast, *Caranx melampygus* recorded no differences with any tested factor when accounting for its absence beyond 53 m across the archipelago. Habitat served to obfuscate the 3-factor design (p > 0.45) for *Carangoides orthogrammus*. When constrained to 53 m or less, depth was significant in the MHI (p < 0.01) as a result of a 6–15 fold increase in abundance between 0–30 m hard-bottom and all substrates between 30–53 m (Supplementary Material Table S2).

**Table 4:** Average abundance (mean *MaxN*) and standard error (SE) of select roving predator species sampled on hard-bottom vs. unconsolidated substrate in the Hawaiian Archipelago.

Species	Main Hawaiian Islands			Northwestern Hawaiian Islands	
	Depth Strata (m)	Hard-bottom	Unconsolidated sediment	Hard-bottom	Unconsolidated sediment
*Aprion virescens*	0–30	0.21 ± 0.07	0.50 ± 0.50	0.74 ± 0.10	na
	30–53	0.57 ± 0.21	0.40 ± 0.40	1.21 ± 0.16	0.33 ± 0.33
	53–100	0.40 ± 0.16	0.54 ± 0.36	0.91 ± 0.15	2.50 ± 1.91
*Caranx melampygus*	0–30	0.79 ± 0.19	—	0.67 ± 0.14	na
	30–53	0.43 ± 0.19	0.80 ± 0.58	1.16 ± 0.47	0.47 ± 1.00
	53–100	—	—	—	—
*Carangoides orthogrammus*	0–30	0.08 ± 0.06	—	0.11 ± 0.08	na
	30–53	0.52 ± 0.24	1.20 ± 0.80	0.63 ± 0.30	—
	53–100	0.28 ± 0.24	0.57 ± .40	—	—
*Caranx ignobilis*	0–30	—	—	1.70 ± 0.70	na
	30–53	0.13 ± 0.07	—	1.20 ± 0.60	—
	53–100	0.10 ± 0.10	0.18 ± 0.08	—	—
*Seriola sp*.*†*	0–30	0.05 ± 0.05	—	0.11 ± 0.08	na
	30–53	0.04 ± 0.04	—	0.47 ± 0.23	—
	53–100	0.40 ± 0.22	0.18 ± 0.15	2.39 ± 1.28	2.50 ± 2.11
*Pseudocaranx cheilio*	0–30	—	—	—	na
	30–53	—	—	0.16 ± 0.04	—
	53–100	—	—	0.43 ± 0.16	4.00 ± 1.63
*Carcharhinus plumbeus*	0–30	—	—	—	na
	30–53	—	—	—	—
	53–100	0.10 ± 0.10	0.43 ± 0.17	0.22 ± 0.09	0.33 ± 0.21
*Carcharhinus galapagensis*	0–30	—	—	0.41 ± 0.17	na
	30–53	—	—	1.26 ± 0.48	—
	53–100	—	—	0.35 ± 0.13	0.83 ± 0.65
*Triaenodon obesus*	0–30	—	—	0.44 ± 0.11	na
	30–53	—	—	0.68 ± 0.23	—
	53–100	—	—	0.04 ± 0.04	—

*Seriola sp* ^†^: pooled totals of *Seriola dumerili*, *Seriola rivoliana*, and unidentified *Seriola sp*.

*Caranx ignobilis* and *Seriola sp* ^†^ (both p = 0.0001, Table 3) were an order of magnitude more abundant in the NWHI (Table 2). In particular, only small numbers of *Caranx ignobolis* were encountered between 30–100 m (upper and lower mesophotic zones) in the MHI (Tables 3–4, Supplementary Material Table S2, and Fig. 5 middle left) in contrast with estimates recorded between 0–53 m in the NWHI. When accounting for dispersion heterogeneity driven by depth absences and habitat obfuscation, pair-wise tests retained regional dissimilarities between counts compared between 30–53 m (Supplementary Materials, Table S2). In contrast, *Seriola sp* ^†^ recorded between 3–8 (MHI) and 21–22 (NWHI) times higher abundances in 53–100 m versus 0–30 m (Tables 3–4, Fig. 5 center). Following the inclusion of pooled habitats, retests for region and depth remained significant (both p = 0.0001), interactive, and heterogeneously dispersed, primarily due to the 6–13 fold abundance increase between 53–100 m in the NWHI (p < 0.001), and asymmetric, intra-regional differences in shallow versus mesophotic strata. Lastly, *Pseudocaranx cheilio* were completely absent in shallow waters and often observed schooling with *Seriola sp* ^†^ in mesophotic depths (Tables 3–4), although no differences were detected between mesophotic zones (Supplementary Materials, Table S2).

The most commonly encountered shark in the MHI - *Carcharhinus plumbeus* - were recorded exclusively in the lower mesophotic zone (Fig. 5, bottom left), with nearly 4 times the number of sightings occurring on unconsolidated sediment compared to hard-bottom substrate with a similar general pattern evident in the NWHI (Table 4). Regional abundances were homogenous and non-significant when pooled habitats were compared between regions (p > 0.05, Supplementary Materials, Table S2). Finally, the two species of shark only recorded in the NWHI - *Carcharhinus galapagensis* and *Triaenodon obesus* - similarly had peak abundances between 30–53 m, were present in 0–30 m, and uncommon in 53–100 m. Despite *Carcharhinus galapagensis* abundance peaking in the upper mesophotic zone (Table 3 and Fig. 5, bottom center), there were no significant depth differences even when habitats were pooled. Similarly, *Triaenodon obesus* was most frequently encountered in the upper mesophotic zone (Fig. 5, lower right), with significant differences between depth strata (p < 0.01, Table 4); However, subsequent PERMDISP comparisons of abundance were homogenously dispersed and non-significant between 0–30 and 30–53 m, coinciding with abundance peaks in those strata and its comparative rarity in deeper depths (Supplementary Materials, Table Results from univariate species-level regression spline models, with depth presented as a continuous variable for each species identified in SIMPER by region, corresponded with previously described patterns. A graphical summary can be found in Supplementary Materials, Figure S2).

### Roving Predator Length Estimates

**Figure 6:**
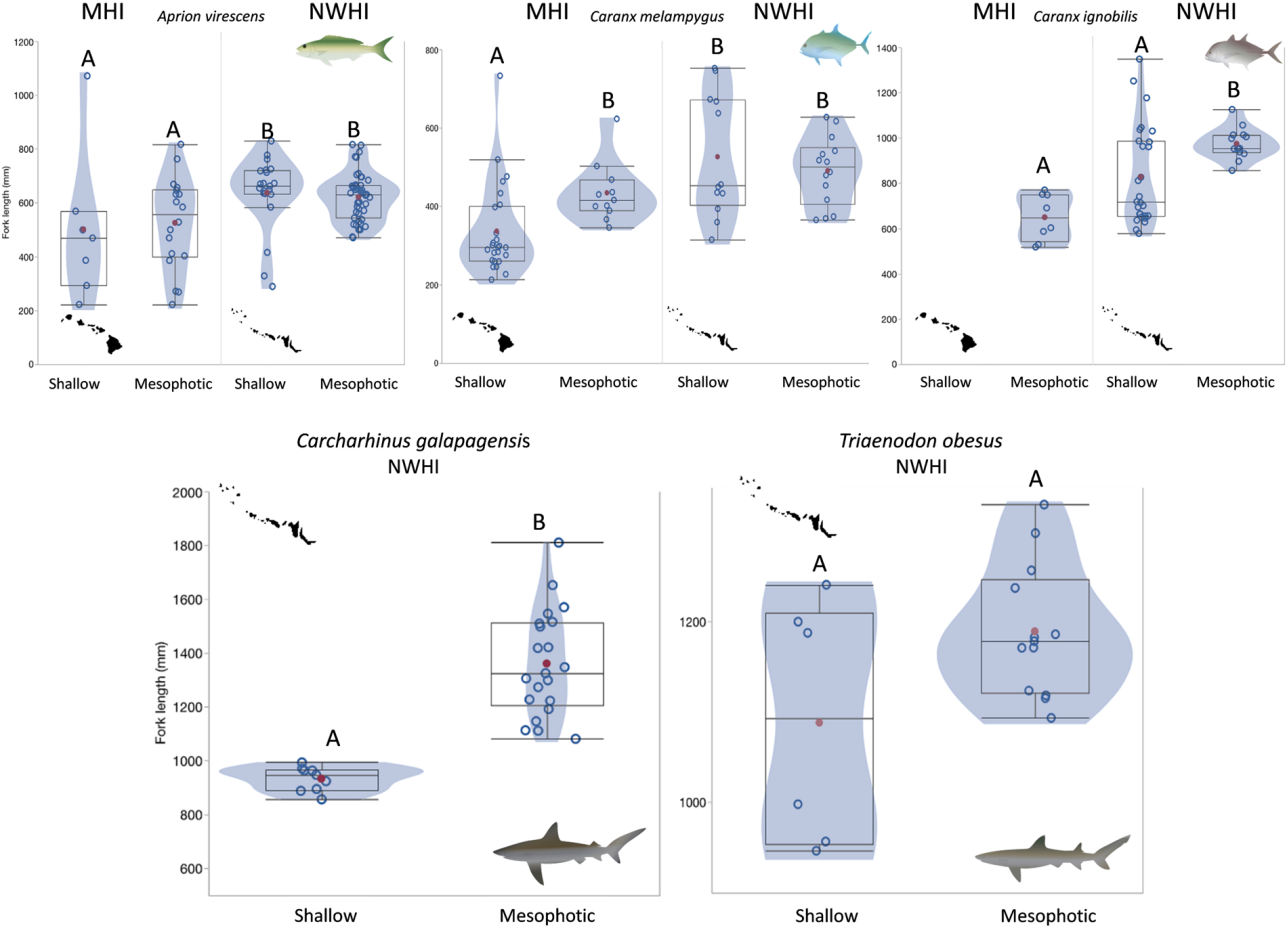
*Box and whisker plots* indicating fork-length size distributions for *Aprion virescens*, *Caranx melampygus*, *Caranx ignobilis*, *Carcharhins galapagensis*, and *Triaenodon obesus*. Whiskers indicate minimum and maximum values, the box specifies the lower interquartile range, and the solid black line indicates the median. Columns with the same letter are not significantly different (P > 0.05). Empty blue circles = individual fork-lengths, solid red circles = mean, shaded contour = density of measurements by length. *Box and whisker* plots generated in JMP statistical software, version 12.1.0 (http://www.jmp.com/). Maps in the figure were generated using Adobe Illustrator CS5 (https://www.adobe.com/products/illustrator.html).

*Aprion virescens* (519 ± 40 and 626 ± 13 mm) and *Caranx melampygus* (367 ± 19 and 507 ± 24, Table 5 and Fig. 6) were significantly smaller (both species, p = 0.0014) in the MHI than in the NWHI. While there were no differences relating to depth strata for *Aprion virescens* (p = 0.5412, Table 6) in either region, *Caranx melampygus* mean size was 29% larger at MHI mesophotic sites than at shallow-water sites in <30 m (435 ± 23 versus 337 ± 23 mm, p = 0.0007). In addition, *Caranx ignobilis* mean size was 26% smaller in the MHI (650 ± 36 mm) than in the NWHI (878 ± 30 mm, p < 0.01, Tables 5 and 6, Fig. 6), primarily driven by larger individuals in the NWHI observed in mesophotic strata. Finally, *Carcharhinus galapagensis* mean size was 45% larger in mesophotic depths compared to shallow in the NWHI (1361 ± 43; 934 ± 15 mm), contrasting with *Triaenodon obesus* which recorded no significant depth-based size differences (1189 ± 20; 1088 ± 55 mm, p > 0.05). Comparisons made with less than 10 measurements (*Aprion virescens*: MHI, 0–30 m and *Triaenodon obesus*: NWHI, 0–30 m) should be treated with caution.

**Table 5:** Mean average length (L_mean_) and standard error (±) for five major roving predator species in Hawaii.

*Species*	MHI Shallow				MHI Mesophotic				MHI Total		NWHI Shallow				NWHI Mesophotic				NWHI Total	
	*n*	*L*_*mean*_	*L*_min_	*L*_max_	*n*	*L*_*mean*_	*L*_min_	*L*_max_	*n*	*L*_*mean*_	*n*	*L*_*mean*_	*L*_min_	*L*_max_	*n*	*L*_*mean*_	*L*_min_	*L*_max_	*n*	*L*_*mean*_
**Snappers** (**Lutjanidae**)																				
*Aprion virescens*	7	502 ± 105	222	1072	18	526 ± 40	222	817	25	519 ± 40	21	638 ± 30	289	830	47	621 ± 13	471	817	68	626 ± 13
**Jacks** (**Carangidae**)																				
*Caranx melampygus*	25	337 ± 23	213	733	11	435 ± 23	346	623	36	367 ± 19	12	527 ± 45	315	752	14	491 ± 23	365	627	26	507 ± 24
*Caranx ignobilis*	—	—	—	—	8	650 ± 36	519	770	8	650 ± 36	27	828 ± 42	578	1348	14	974 ± 18	857	1126	41	878 ± 30
**Sharks** (**Carcharhinidae**)																				
*Carcharhinus galapagensis*	—	—	—	—	—	—	—	—	—	—	9	934 ± 15	857	994	21	1361 ± 43	1082	1810	30	1233 ± 47
*Triaenodon obesus*	—	—	—	—	—	—	—	—	—	—	6	1088 ± 55	946	1241	13	1189 ± 20	1093	1330	19	1157 ± 19

Minimum (L_min_) and maximum (L_min_) lengths are noted for each species, within each depth strata (shallow, mesophotic) and region (MHI, NWHI).

**Table 6:** Results of Kolmogorov-Smirnov (K-S) tests of differences between pairs of fish length density distributions sampled by region and depth strata.

*Aprion virescens*			*Caranx melampygus*		*Caranx ignobilis*		*Carcharhinus galapagensis*		*Triaenodon obesus*	
Region, Depth Strata	D Statistic	P	D Statistic	P	D Statistic	P	D Statistic	P	D Statistic	P
MHI, NWHI (Totals)	0.4465	**0**.**0014**	0.4915	**0**.**0014**	0.6341	**0**.**0092**	—	—	—	—
MHI 0–30 m, MHI 30–100 m	0.3571	0.5412	0.7200	**0**.**0007**	—	—	—	—	—	—
MHI 0–30 m, NWHI 0–30 m	0.7143	**0**.**0094**	0.6400	**0**.**0026**	—	—	—	—	—	—
MHI 0–30 m, NWHI 30–100 m	0.6717	**0**.**0082**	0.7200	**0**.**0002**	—	—	—	—	—	—
MHI 30–100 m, NWHI 0–30 m	0.4841	**0**.**0213**	0.4167	0.2719	0.4444	0.1745	—	—	—	—
MHI 30–100 m, NWHI 30–100 m	0.4019	**0**.**0299**	0.4610	0.1457	1.0000	**<0**.**0001**	—	—	—	—
NWHI 0–30 m, NWHI 30–100 m	0.3202	0.1020	0.4167	0.2119	0.6296	**0**.**0013**	1.0000	**<0**.**0001**	0.5000	0.2562

Bonferroni corrections were applied to for multiple depth comparisons (*Aprion virescens* and *Caranx melampygus*, α = 0.0083; *Caranx ignobilis*, α = 0.017). Values in bold are significant at <0.05.

### Habitat Description and Predator Linkages

Environmental variables were similar between shallow and upper mesophotic zones PCA ordinations in the MHI and NWHI (Figure S3A–C). However, DISTLM-dbRDA linkages between discriminant MHI roving predators (Pearson’s r > 0.25, *Caranx melampygus*, *Carcharhinus plumbeus*, *Seriola sp* ^†^) and environmental variables were weakly correlated, with only 10.5% of the total variation accounted for by depth, % turf algae, and habitat complexity (Figure S3D). While several NWHI species aligned with areas of greater habitat complexity (e.g. *Carcharhinus galapagensis*, *Caranx ignobilis*), and depth (*Seriola sp*., *Carcharhinus plumbeus*),DISTLM-dbRDA linkages remained weak with only 18.6% of the variation explained by % hard coral, % macroalgae, habitat complexity, and depth. More detail is given in Supplementary Materials, Figure S3C,D.

## Discussion

For assessing predator populations, BRUVS offer several potential benefits over shallow water diver surveys. Aside from removing depth constraints associated with open-circuit scuba and potential bias due to different responses of fishes to divers in different locations, i.e. predator avoidance in populated areas and attraction in remote areas^80–82^, and reducing concerns associated with diver instantaneous versus non-instantaneous predator counts^6^, archived video can be used to extract data on other species or to verify the authenticity of predator identifications and length measurements^83^. Like all field survey methods, BRUVS have limitations including deployment challenges in vertical habitats, variable bait plume areas^48, 84^, and the potential for competitive exclusion of some species^50, 84, 85^. However, for roving reef predators that are often rare or absent during underwater visual censuses, BRUVS may provide a better community-wide representation of assemblage composition^34, 48, 50^.

Survey results were consistent with predator abundance patterns documented in underwater visual censuses in the MHI and NWHI, albeit over a wider depth range (0–100 m). While pooled abundance values (all species) were three times higher in the NWHI (Table 2), differences were more pronounced for gregarious species. Specifically, *Caranx ignobilis* and pooled *Seriola sp* ^*†*^ were over an order of magnitude more abundant in the NWHI (all depths and habitats combined), which aligns with historic predator densities recorded by belt transect in ≤30 m^−1^, although reported belt-derived ratios for *Caranx ignoblis* alone were considerably higher than 10:1.

**Figure 7:**
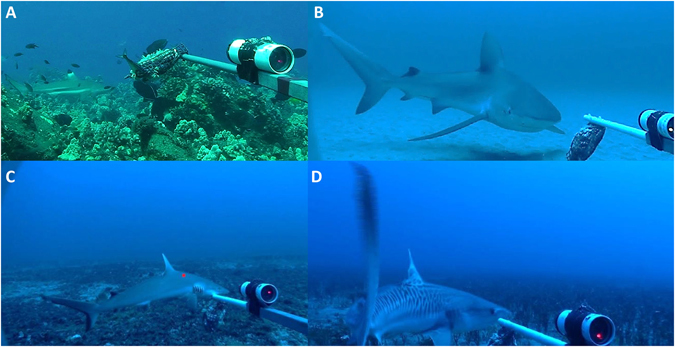
(**A**) *Carcharhinus melanopterus*, Lanai. (**B**) *Carcharhinus limbatus*, Oahu. Mesophotic sighting outside of 1-hour BRUVS sampling period (**C**) C*archarhinus amblyrhynchos*, Maui. (**D**) Juvenile *Galeocerdo cuvier*, Maui. BRUVS frame grab photographs by J. Asher.

Sharks can be patchily distributed over fine spatial scales^86, 87^, and while BRUVS sampled more shark species than are typically encountered by open-circuit scuba divers, several were potentially underrepresented (or were not recorded at all) in this study. This likely came as a result of (1) sparse sampling or exclusion of some habitat types (e.g. backreef and lagoons were not sampled) and/or several Hawaiian islands, (2) constraints due to limited seasonal and day-time only sampling; and (3) one-hour BRUVS soak time limits. For example, only a single blacktip reef shark was sighted during MHI surveys (*Carcharhinus melanopterus*, shallow water observation at Lanai, Fig. 7A), but localized aggregations of that species are known to occur, e.g. at Pelekane Bay on the Big Island, MHI^88^. In addition, a single mesophotic blacktip shark (*Carcharhinus limbatus*) sighting occurred outside of the one-hour BRUVS sampling period on Oahu (Fig. 7B) and while both species can be found in low numbers in the both the MHI and NWHI, their absence during this study suggests future BRUVS sampling could be improved, at minimum, by expanding surveys to include additional islands in the Hawaiian Archipelago, increasing the number of sites at each island, and incorporating backreef/lagoonal environs into future designs^31, 89^.

Similarly, no *Carcharhinus galapagensis* or *Triaenodon obsesus* were sampled during MHI BRUVS surveys, and only two *Carcharhinus amblyrhynchos* were recorded (one mesophotic site off of the Maui town of Lahaina (Fig. 7C), and one shallow water site off of the south shore of Oahu). While Galapagos sharks were once deemed abundant in the MHI and are noted for frequent sightings and seasonal movements in certain areas (e.g. north shore of Oahu), they remain spatially restricted around islands hosting high human population densities, with historic catch-rates low in comparison with sandbar, tiger, and grey reef sharks^89–94^. In addition, while MHI divers on open-circuit scuba may encounter grey reef sharks or white tip reef sharks more frequently than other species, they appear relatively uncommon and/or patchily distributed outside of localized populations, with known grey reef shark aggregations around Molokini, Niihau, and Ka’ula Rock in the MHI and Necker and French Frigate Shoals in the NWHI, and historic white tip reef shark sightings along Oahu’s western, southern, and eastern shorelines, along with South Maui, Molokini, and the Kona coast of the Big Island^91, 95, 96^. When combining the absence (in BRUVS surveys) of both species in the MHI irrespective of surveyed depths or habitats, scant grey reef and blacktip reef shark sightings, common bycatch rates observed for commercial and recreational fisheries across the state’s coastal waters, and the 5-fold difference in pooled shark abundances between MHI and NWHI (Table 2), these collectively serve as additional evidence towards reduced reef-shark baselines around populated areas^7, 95–97^.

The majority of MHI BRUV sightings occurred in mesophotic depths. Aside from *Carcharhinus plumbeus* and a single *Carcharhinus amblyrhynchos*, these consisted exclusively of mature or small-bodied (≤2 m) female tiger sharks, *Galeocerdo cuvier* (Fig. 7D), which may be indicative of migratory patterns documented from the NWHI to the MHI linked to the September – November pupping season, or possible evidence of sex segregation^98, 99^. While additional environmental effects (thermal, available food resources) may explain the increased mesophotic presence seen here, interpretations based on small BRUVS sample sizes should be treated with caution, as tiger sharks are depth-generalists that may be considerably more abundant (comprising up to 20% of all sharks captured during longline surveys in the NWHI) than accounted for in this study^17, 31^.

Overlaps between shallow water and upper mesophotic zone roving predator communities, coupled with a partial separation of lower mesophotic zone assemblages, was seen in both the MHI and NWHI, albeit with divergent species and depth distributions driving interzone connectivity. In the NWHI, *Aprion virescens* remained more broadly distributed between depth strata; however, shallow water-upper mesophotic zone overlaps for three jacks (*Caranx melampygus*, *Caranx ignobilis*, and *Carangoides orthogrammus*) and the numeric majority of two sharks (*Triaenodon obesus* and *Carcharhinus galapagensis*) between 0–53 m hint at several possible, interactive drivers, including prey-partitioning mechanisms^100^, competition with more abundant species in the lower mesophotic zone (e.g. *Seriola sp* ^†^, *Carcharhinus plumbeus*), and/or the reduced density of preferred prey in deeper depths. While isotopic analyses indicate *Carcharhinus galapagensis* primarily forage in shallow water, their movements in mesophotic depths may be underestimated, and runs contrary to longline studies which captured the majority of *Carcharhinus galapagensis* between 40–45 m^15, 29, 92^. The prevalence of smaller *Carcharhinus galapagensis* and *Caranx ignobilis* in <30 m depths suggests possible body size and depth segregation, potential avoidance of intra- or inter-specific predation pressures^101^ in deeper waters despite documented juvenile Galapagos shark movements in mesophotic depths thought to be tied to diel, vertical migration patterns, and no evidence of NWHI shallow water nursery areas as seen elsewhere^91, 92, 102^. Predator alignments with thermocline position^103^, and increases in mesophotic fish densities (i.e. prey availability) between 50–60 m^25, 73^ coincide with higher upper mesophotic abundances documented for the principal species encountered during NWHI BRUVS surveys. Finally, Hawaiian monk seal (*Neomonachus schauinslandi*) Crittercam^TM^ surveys noted peak predator escort and foraging interactions between seals and *Aprion virescens*, sharks, and jacks occurring between 60–80 m, suggesting predator depth adjustments may be coupled to seal foraging in some cases^30, 104^. The general absence of predator movements in >100 m depths or interisland transits^15, 30, 105, 106^ are indicative of predatory spatial residency, and that BRUVS appear to capture overall depth-range demographics for these aforementioned species in the NWHI. This appears to be confirmed with historic bait station and submersible surveys in bottom fish depths (286–657 m), where shark sightings were rare and *Aprion virescens*, *Caranx ignobilis*, *Caranx melampygus*, and *Carangoides orthogrammus* were absent, but *Pseudocaranx cheilio* and *Seriola sp* ^*†*^ were commonly sighted^107^. Whether size-differences or depth distributions directly relate to prey-partitioning, proportionately available habitats and host prey resources, competition with other species in deeper depths, or other causes remains an important area for future research, along with expanded investigations into Carangidae diel, lunar, and seasonal migrations (e.g. with *Caranx ignobilis*)^105^, and nutrient transport potential between depth zones^15^.

Large-bodied snappers, jacks and sharks are susceptible to fishing activities in the MHI, and changes in abundance and/or biomass may be indicative of extraction pressures^12, 25, 108, 109^. Mesophotic habitats may act as depth-refuges for species considered particularly vulnerable to fishing^16, 25, 110^, and evidence from this study remains suggestive of potential depth insulation for several predators in the MHI, mirroring patterns seen elsewhere^26, 39, 103, 111^. In particular, *Caranx melampygus* was one of the primary species responsible for shallow-upper mesophotic zone overlaps in the MHI, with relatively similar numbers recorded between zones; however, overall mean fork-lengths were smaller in <30 m than at mesophotic sites or at sites in the NWHI. *Carangoides orthogrammus* were 6–15 times more abundant in the upper mesophotic zone than in diver depths, and *Caranx ignobilis* were only recorded in mesophotic zones in the MHI. In contrast, inferences on MHI shark population parameters are limited by low number of encounters during this study. Sightings of *Carcharhinus plumbeus* align with previous research, which have shown sandbar sharks to be the most common shark species in the MHI, that they are primarily captured in 60–90 m depths (although they may diurnally migrate to shallower depths of 18–20 m at night, and depth-segregate by age and sex), and that they are less abundant than several other shark species at location in the NWHI^91, 112^. However, in this study, mean abundance (0.12 ± 0.05 vs. 0.09 ± 0.03) and encounter rate (8.4% vs. 9%) were similar between regions (Table 2, which coupled with comparable longline catch-rates at French Frigate Shoals^31^, suggests that sandbar sharks may not be as uncommon in the NWHI as previously suspected.

Pooled environmental covariates delineated largely along *a priori* designated survey depth strata, with overlaps between regions, i.e. environmental variables generally appear similar between the MHI vs. NWHI (Supplementary Materials, Figure S3A–C). However, environmental linkages with roving predator assemblages were tenuous at best (Supplementary Materials, Figure S3D,E), and may be indicative of (a) the highly mobile nature of the roving predators and the utilization of multiple habitats; or (b) limited or asymmetric sampling frequencies between depths and habitats.

Finally, most open-circuit dive surveys focus exclusively on hard-bottom substrates, which may miss a proportion of the predator population occupying large areas of unconsolidated sediment in the Hawaiian Archipelago (especially the MHI). While roving predators may retain inherent preferences towards hard-bottom substrates, the assessed species presented here (except for whitetip reef sharks) are known to utilize shallow water sandy habitats^13, 105, 106, 113–115^ and were similarly encountered on mesophotic sand flats during the course of this study. In addition, *Caranx melampygus*, *Caranx ignobilis*, *Aprion virescens*, and *Seriola sp* ^†^ were all observed feeding in areas of unconsolidated sediment (J. Asher, pers. obs.); however, while several studies indicate the presence of high predator biomass over sand flats in comparison with other functional groups, the frequency and ecological effects of sand flat usage as foraging grounds, seasonal aggregation sites, refugia, or as transitional habitats (i.e. as corridors between areas hosting higher complexity, hard-bottom substrates) remains largely unaccounted for^97, 116, 117^. Future BRUVS surveys would benefit from the inclusion of these areas in subsequent designs, as roving predators normally associated with reef and hard-bottom systems are clearly present in deeper, underexplored, unconsolidated sediment habitats in the MHI.

In conclusion, roving predator research has been heavily reliant on underwater visual censuses, along with a smaller number of fishery independent remote underwater video surveys, tracking studies, and limited fishery-dependent or extractive surveys^31^. The use of BRUVS and the expansion of surveys into mesophotic depths augment our understanding of roving predator distributions across the Hawaiian Archipelago, and illustrate the need to expand long-term predator research and monitoring beyond open-circuit SCUBA depths.

## Electronic supplementary material

Supplementary Materials

## References

[1] Friedlander AM, DeMartini EE (2002). Contrasts in density, size, and biomass of reef fishes between the northwestern and the main Hawaiian islands: the effects of fishing down apex predators. Mar Ecol-Prog Ser.

[2] Baum JK (2003). Collapse and conservation of shark populations in the Northwest Atlantic. Science.

[3] Myers RA, Baum JK, Shepherd TD, Powers SP, Peterson CH (2007). Cascading effects of the loss of apex predatory sharks from a coastal ocean. Science.

[4] Baum JK, Myers RA (2004). Shifting baselines and the decline of pelagic sharks in the Gulf of Mexico. Ecol Lett.

[5] Dulvy NK (2008). You can swim but you can’t hide: the global status and conservation of oceanic pelagic sharks and rays. Aquatic Conservation: Marine and Freshwater Ecosystems.

[6] Ward-Paige CA (2010). Large-Scale Absence of Sharks on Reefs in the Greater-Caribbean: A Footprint of Human Pressures. Plos One.

[7] Nadon MO (2012). Re-Creating Missing Population Baselines for Pacific Reef Sharks. Conserv Biol.

[8] Santos SR, Xiang Y, Tagawa AW (2011). Population structure and comparative phylogeography of jack species (Caranx ignobilis and C. melampygus) in the high Hawaiian Islands. J Hered.

[9] Randall, J. E. *Reef and shore fishes of the Hawaiian Islands*. (Sea Grant College Program, University of Hawai’i, 2007).

[10] Nadon MO, Ault JS, Williams ID, Smith SG, DiNardo GT (2015). Length-based assessment of coral reef fish populations in the Main and northwestern Hawaiian islands. Plos One.

[11] Friedlander, A. & Dalzell, P. In *Status of Hawaii’s Coastal Fisheries in the New Millennium, revised 2004 edition. Proceedings of the 2001 fisheries symposium sponsored by the American Fisheries Society, Hawai’i Chapter*. 171–185 (Citeseer).

[12] Williams, I. D. *et al*. Differences in Reef Fish Assemblages between Populated and Remote Reefs Spanning Multiple Archipelagos Across the Central and Western Pacific. *Journal of Marine Biology* **2011**, doi:10.1155/2011/826234 (2011).

[13] Holzwarth SR, DeMartini EE, Schroeder R, Zgliczynski B, Laughlin J (2006). Sharks and jacks in the Northwestern Hawaiian Islands from towed-diver surveys 2000–2003. Atoll Res Bull.

[14] Pickard, A. E. *Characterization of Shark Movements on a Mesophotic Caribbean Coral Reef and Temporal Association with Fish Spawning Aggregations*. Master’s thesis, Nova Southeastern University, Oceanographic Center (2013).

[15] Papastamatiou YP, Meyer CG, Kosaki RK, Wallsgrove NJ, Popp BN (2015). Movements and foraging of predators associated with mesophotic coral reefs and their potential for linking ecological habitats. Mar Ecol Prog Ser.

[16] Bejarano I, Appeldoorn R, Nemeth M (2014). Fishes associated with mesophotic coral ecosystems in La Parguera, Puerto Rico. Coral Reefs.

[17] Holland K, Wetherbee B, Lowe C, Meyer C (1999). Movements of tiger sharks (Galeocerdo cuvier) in coastal Hawaiian waters. Mar Biol.

[18] Nakamura I, Watanabe YY, Papastamatiou YP, Sato K, Meyer CG (2011). Yo-yo vertical movements suggest a foraging strategy for tiger sharks Galeocerdo cuvier. Mar Ecol Prog Ser.

[19] Meyer CG, Papastamatiou YP, Clark TB (2010). Differential movement patterns and site fidelity among trophic groups of reef fishes in a Hawaiian marine protected area. Mar Biol.

[20] Fitzpatrick R (2012). A comparison of the seasonal movements of tiger sharks and green turtles provides insight into their predator-prey relationship. Plos One.

[21] Werry JM (2014). Reef-fidelity and migration of tiger sharks, Galeocerdo cuvier, across the Coral Sea. Plos One.

[22] Randall JE (1977). Contribution to the biology of the whitetip reef shark (Triaenodon obesus). Pac Sci.

[23] Ralston S, Gooding RM, Ludwig GM (1986). An ecological survey and comparison of bottom fish resource assessments (submersible versus handline fishing) at Johnston Atoll. Fish B-Noaa.

[24] Chave, E. & Mundy, B. Deep-sea benthic fish of the hawaiian Archipelago, Cross Seamount, and Johnston Atoll. *Pac Sci* (1994).

[25] Lindfield, S. J., Harvey, E. S., Halford, A. R. & McIlwain, J. L. Mesophotic depths as refuge areas for fishery-targeted species on coral reefs. *Coral Reefs* 1–13 (2016).

[26] Bongaerts P, Ridgway T, Sampayo EM, Hoegh-Guldberg O (2010). Assessing the ‘deep reef refugia’ hypothesis: focus on Caribbean reefs. Coral Reefs.

[27] Bridge TC, Hughes TP, Guinotte JM, Bongaerts P (2013). Call to protect all coral reefs. Nature Climate Change.

[28] Kahng S, Copus J, Wagner D (2014). Recent advances in the ecology of mesophotic coral ecosystems (MCEs). Current Opinion in Environmental Sustainability.

[29] Meyer CG, Papastamatiou YP, Holland KN (2010). A multiple instrument approach to quantifying the movement patterns and habitat use of tiger (Galeocerdo cuvier) and Galapagos sharks (Carcharhinus galapagensis) at French Frigate Shoals, Hawaii. Mar Biol.

[30] Parrish FA, Marshall GJ, Buhleier B, Antonelis GA (2008). Foraging interaction between monk seals and large predatory fish in the Northwestern Hawaiian Islands. Endangered Species Research.

[31] Dale, J. J., Stankus, A. M., Burns, M. S. & Meyer, C. G. The Shark Assemblage at French Frigate Shoals Atoll, Hawai’i: Species Composition, Abundance and Habitat Use. *Plos One* **6**, doi:10.1371/journal.pone.0016962 (2011). 10.1371/journal.pone.0016962 PMC3037392 21347321

[32] Zintzen V (2012). Diversity and composition of demersal fishes along a depth gradient assessed by baited remote underwater stereo-video. Plos One.

[33] Goetze J, Fullwood L (2013). Fiji’s largest marine reserve benefits reef sharks. Coral Reefs.

[34] Brooks EJ, Sloman KA, Sims DW, Danylchuk AJ (2011). Validating the use of baited remote underwater video surveys for assessing the diversity, distribution and abundance of sharks in the Bahamas. Endangered Species Research.

[35] Espinoza M, Cappo M, Heupel MR, Tobin AJ, Simpfendorfer CA (2014). Quantifying Shark Distribution Patterns and Species-Habitat Associations: Implications of Marine Park Zoning. Plos One.

[36] Rizzari JR, Frisch AJ, Connolly SR (2014). How robust are estimates of coral reef shark depletion?. Biol Conserv.

[37] Santana‐Garcon J (2014). Calibration of pelagic stereo‐BRUVs and scientific longline surveys for sampling sharks. Methods in Ecology and Evolution.

[38] Malcolm HA, Schultz AL, Sachs P, Johnstone N, Jordan A (2015). Decadal Changes in the Abundance and Length of Snapper (Chrysophrys auratus) in Subtropical Marine Sanctuaries. Plos One.

[39] Pinheiro H (2016). Upper and lower mesophotic coral reef fish communities evaluated by underwater visual censuses in two Caribbean locations. Coral Reefs.

[40] Williams ID (2015). Human, Oceanographic and Habitat Drivers of Central and Western Pacific Coral Reef Fish Assemblages. Plos One.

[41] Ayotte, P., McCoy, K., Heenan, A., Williams, I. & Zamzow, J. Coral Reef Ecosystem Program Standard Operating Procedures: Data Collection for Rapid Ecological Assessment Fish Surveys. *Pacific Islands Fisheries Science Center Administrative Report H*-*15*-*07*, *33p* (2015).

[42] Harvey E, Shortis M, Stadler M, Cappo M (2002). A comparison of the accuracy and precision of measurements from single and stereo-video systems. Mar Technol Soc J.

[43] Harvey E, Shortis M (1995). A system for stereo-video measurement of sub-tidal organisms. Mar Technol Soc J.

[44] Harvey ES, Shortis MR (1998). Calibration stability of an underwater stereo-video system: Implications for measurement accuracy and precision. Mar Technol Soc J.

[45] Seager, J. SEAGIS CAL and Photomeasure stereo photogrametric calibration and analysis software, http://www.seagis.com.au (2008).

[46] Harvey, E. *et al*. The use of BRUVs as a tool for assessing marine fisheries and ecosystems: a review of the hurdles and potential (2011 National Workshop). *Fisheries Research and Development Corporation and The University of Western Australia* Project No. 2010/002, ISSN: 978-971-74052-74265-74056 (2013).

[47] Priede I, Ragley P, Smith K (1994). Seasonal change in activity of abyssal demersal scavenging grenadiers Coryphaenoides (Nematonums) armatus in the eastern North Pacific Ocean. Limnol Oceanogr.

[48] Cappo M, Speare P, De’ath G (2004). Comparison of baited remote underwater video stations (BRUVS) and prawn (shrimp) trawls for assessments of fish biodiversity in inter-reefal areas of the Great Barrier Reef Marine Park. J Exp Mar Biol Ecol.

[49] Cappo, M., Harvey, E., Malcolm, H. & Speare, P. Potential of video techniques to monitor diversity, abundance and size of fish in studies of marine protected areas. *World Congress on Aquatic Protected Areas*, 455–464 (2003).

[50] Willis TJ, Babcock RC (2000). A baited underwater video system for the determination of relative density of carnivorous reef fish. Mar Freshwater Res.

[51] Willis TJ, Millar RB, Babcock RC (2000). Detection of spatial variability in relative density of fishes: comparison of visual census, angling, and baited underwater video. Mar Ecol-Prog Ser.

[52] Ellis DM, Demartini EE (1995). Evaluation of a Video Camera Technique for Indexing Abundances of Juvenile Pink Snapper, Pristipomoides-Filamentosus, and Other Hawaiian Insular Shelf Fishes. Fish B-Noaa.

[53] Harvey ES, Goetze J, McLaren B, Langlois T, Shortis MR (2010). Influence of range, angle of view, image resolution and image compression on underwater stereo-video measurements: high-definition and broadcast-resolution video cameras compared. Mar Technol Soc J.

[54] Pacific Islands Fisheries Science Center, C. R. E. P. *Baited and Unbaited Remote Underwater stereo*-*video* (*BRUV*) *Surveys of Fish at Select U*.*S*. *Pacific Reefs Since 2010*, https://inport.nmfs.noaa.gov/inport/item/25248 (2015).

[55] Wilson S, Graham N, Polunin N (2007). Appraisal of visual assessments of habitat complexity and benthic composition on coral reefs. Mar Biol.

[56] Heenan, A. *et al*. Pacific Reef Assessment and Monitoring Program - Data Report - Ecological Monitoring 2012–2013 - reef fishes and benthic habitats of the main Hawaiian Islands, American Samoa, and Pacific Remote Island Areas. 112 (Pacific Islands Fisheries Science Center, 2014).

[57] Rosa MR (2016). Mesophotic reef fish assemblages of the remote St. Peter and St. Paul’s Archipelago, Mid-Atlantic Ridge, Brazil. Coral Reefs.

[58] Clarke KR, Somerfield PJ, Chapman MG (2006). On resemblance measures for ecological studies, including taxonomic dissimilarities and a zero-adjusted Bray-Curtis coefficient for denuded assemblages. J Exp Mar Biol Ecol.

[59] Anderson, M., Gourley, R. N. & Clarke, K. R. *PERMANOVA*+ *for PRIMER*: *guide to software and statistical methods*. (PRIMER-E, 2008).

[60] Clarke, K. & Gorley, R. *PRIMER v7*: *User Manual/Tutorial* 296 pp (2015).

[61] Efron, B. *The jackknife*, *the bootstrap and other resampling plans*. Vol. 38 (SIAM, 1982).

[62] Manly, B. F. *Randomization*, *bootstrap and Monte Carlo methods in biology*. Vol. 70 (CRC Press, 2006).

[63] Cox, T. F. & Cox, M. A. *Multidimensional scaling*. (CRC Press, 2000).

[64] Rousseeuw PJ (1987). Silhouettes: a graphical aid to the interpretation and validation of cluster analysis. Journal of computational and applied mathematics.

[65] Rohlf, F. J. & Wooten, M. C. Evaluation of the restricted maximum-likelihood method for estimating phylogenetic trees using simulated allele-frequency data. *Evolution* 581–595 (1988). 10.1111/j.1558-5646.1988.tb04162.x 28564007

[66] Anderson MJ, Willis TJ (2003). Canonical analysis of principal coordinates: a useful method of constrained ordination for ecology. Ecology.

[67] Anderson MJ, Robinson J (2003). Generalized discriminant analysis based on distances. Aust Nz J Stat.

[68] Anderson MJ, Walsh DC (2013). PERMANOVA, ANOSIM, and the Mantel test in the face of heterogeneous dispersions: What null hypothesis are you testing?. Ecological monographs.

[69] Wilkinson L, Friendly M (2009). The history of the cluster heat map. The American Statistician.

[70] Clarke KR, Somerfield PJ, Gorley RN (2008). Testing of null hypotheses in exploratory community analyses: similarity profiles and biota-environment linkage. J Exp Mar Biol Ecol.

[71] Clarke, K. R. Nonparametric Multivariate Analyses of Changes in Community Structure. *Aust J Ecol* **18**, 117–143 (1993).

[72] Clarke, K. & Warwick, R. Change in Marine Communities: An Approach to Statistical Analysis and Interpretation. *2nd Edition*.(*PRIMER*-*E Ltd*: *Plymouth*, *United Kingdom*) (2001).

[73] Fukunaga A, Kosaki RK, Wagner D, Kane C (2016). Structure of Mesophotic Reef Fish Assemblages in the Northwestern Hawaiian Islands. Plos One.

[74] Anderson MJ (2008). Animal-sediment relationships re-visited: Characterising species’ distributions along an environmental gradient using canonical analysis and quantile regression splines. J Exp Mar Biol Ecol.

[75] R Core Team. R: A language and environment for statistical computing, vers. 3.3.0. R Foundation for Statistical Computing, Vienna, Austria. URL http://www.R-project.org/ (2016).

[76] Massey FJ (1951). The Kolmogorov-Smirnov test for goodness of fit. Journal of the American statistical Association.

[77] Jolliffe, I. *Principal component analysis*. (Wiley Online Library, 2002).

[78] Wold S, Esbensen K, Geladi P (1987). Principal component analysis. Chemometrics and intelligent laboratory systems.

[79] Anderson, M. J. DISTLM v. 5: A FORTRAN computer program to calculate a distance-based multivariate analysis for a linear model. *Department of Statistics*, *University of Auckland*, *New Zealand* **10** (2004).

[80] Bozec YM, Kulbicki M, Laloe F, Mou-Tham G, Gascuel D (2011). Factors affecting the detection distances of reef fish: implications for visual counts. Mar Biol.

[81] Thresher RE, Gunn JS (1986). Comparative analysis of visual census techniques for highly mobile, reef-associated piscivores (Carangidae). Environ Biol Fish.

[82] Lindfield SJ, McIlwain JL, Harvey ES (2014). Depth refuge and the impacts of SCUBA spearfishing on coral reef fishes. Plos One.

[83] Cappo, M., Harvey, E. & Shortis, M. Counting and measuring fish with baited video techniques-an overview. *Australian Society for Fish Biology Workshop Proceedings*, Hobart, Tasmania, Australia, 101–114 (August 28–29, 2006).

[84] Stobart B (2007). A baited underwater video technique to assess shallow-water Mediterranean fish assemblages: Methodological evaluation. J Exp Mar Biol Ecol.

[85] Bailey DM, Priede IG (2002). Predicting fish behaviour in response to abyssal food falls. Mar Biol.

[86] Heupel MR, Simpfendorfer CA, Collins AB, Tyminski JP (2006). Residency and movement patterns of bonnethead sharks, Sphyrna tiburo, in a large Florida estuary. Environ Biol Fish.

[87] Grubbs RD, Musick JA, Conrath CL, Romine JG (2007). Long-term movements, migration, and temporal delineation of a summer nursery for juvenile sandbar sharks in the Chesapeake Bay region. American Fisheries Society Symposium.

[88] Hoover, D. J. & Gold, C. Assessment of coastal water resources and watershed conditions at Pu’ukohola Heiau National Historic Site, Hawai ‘i. *National Park Service*, *Technical Report NPS/NRWRD/NRTR*-*2006/359*, 2–133 (2006).

[89] Dale, J. J., Meyer, C. G. & Clark, C. E. The ecology of coral reef top predators in the Papahānaumokuākea Marine National Monument. *Journal of Marine Biology* **2011** (2010).

[90] Meyer CG, Dale JJ, Papastamatiou YP, Whitney NM, Holland KN (2009). Seasonal cycles and long-term trends in abundance and species composition of sharks associated with cage diving ecotourism activities in Hawaii. Environ Conserv.

[91] Papastamatiou YP, Wetherbee BM, Lowe CG, Crow GL (2006). Distribution and diet of four species of carcharhinid shark in the Hawaiian Islands: evidence for resource partitioning and competitive exclusion. Mar Ecol-Prog Ser.

[92] Wetherbee BM, Crow GL, Lowe CG (1996). Biology of the Galapagos shark, Carcharhinus galapagensis, in Hawai’i. Environ Biol Fish.

[93] Wetherbee BM, Lowe CG, Crow GL (1994). A review of shark control in Hawaii with recommendations for future research. Pac Sci.

[94] Tester, A. Cooperative Shark Research and Control Program. *Final Report*, University of Hawaii 1–36 (1969).

[95] Wetherbee BM, Crow GL, Lowe CG (1997). Distribution, reproduction and diet of the gray reef shark Carcharhinus amblyrhynchos in Hawaii. Mar Ecol Prog Ser.

[96] Whitney NM, Pyle RL, Holland KN, Barcz JT (2012). Movements, reproductive seasonality, and fisheries interactions in the whitetip reef shark (Triaenodon obesus) from community-contributed photographs. Environ Biol Fish.

[97] Filous A (2017). Movement patterns of reef predators in a small isolated marine protected area with implications for resource management. Mar Biol.

[98] Papastamatiou YP (2013). Telemetry and random‐walk models reveal complex patterns of partial migration in a large marine predator. Ecology.

[99] Meyer CG, Clark TB, Papastamatiou YP, Whitney NM, Holland KN (2009). Long-term movement patterns of tiger sharks Galeocerdo cuvier in Hawaii. Mar Ecol Prog Ser.

[100] Meyer CG, Holland KN, Wetherbee BM, Lowe CG (2001). Diet, resource partitioning and gear vulnerability of Hawaiian jacks captured in fishing tournaments. Fish Res.

[101] Compagno, L. J. *Sharks of the world*: *an annotated and illustrated catalogue of shark species known to date*. Vol. 2 (Food & Agriculture Org., 2001).

[102] Kato, S. & Carvallo, A. In *Sharks*, *Skates*, *and Rays* Ch. Shark tagging in the eastern Pacific Ocean, 93–109 (Johns Hopkins Press, 1967).

[103] Thresher RE, Colin PL (1986). Trophic structure, diversity and abundance of fishes of the deep reef (30–300 m) at Enewetak, Marshall Islands. B Mar Sci.

[104] Parrish FA (2006). Precious corals and subphotic fish assemblages. Atoll Research Bulletin.

[105] Meyer, C. G., Holland, K. N. & Papastamatiou, Y. P. Seasonal and diel movements of giant trevally Caranx ignobilis at remote Hawaiian atolls: implications for the design of marine protected areas. *Mar Ecol Prog Ser* **333** (2007).

[106] Meyer CG, Papastamatiou YP, Holland KN (2007). Seasonal, diel, and tidal movements of green jobfish (Aprion virescens, Lutjanidae) at remote Hawaiian atolls: implications for marine protected area design. Mar Biol.

[107] Kelley C, Ikehara W (2006). The impacts of bottomfishing on Raita and west St. Rogatien banks in the Northwestern Hawaiian Islands. Atoll Res Bull.

[108] Jennings, S. & Polunin, N. Effects of fishing effort and catch rate upon the structure and biomass of Fijian reef fish communities. *J Appl Ecol* 400–412 (1996).

[109] Weijerman M, Fulton EA, Parrish FA (2013). Comparison of coral reef ecosystems along a fishing pressure gradient. Plos One.

[110] Riegl B, Piller WE (2003). Possible refugia for reefs in times of environmental stress. International Journal of Earth Sciences.

[111] Feitoza BM, Rosa RS, Rocha LA (2005). Ecology and zoogeography of deep-reef fishes in northeastern Brazil. B Mar Sci.

[112] McElroy WD (2006). Food habits and ontogenetic changes in the diet of the sandbar shark, Carcharhinus plumbeus, in Hawaii. Environ Biol Fish.

[113] Smith G, Parrish J (2002). Estuaries as nurseries for the jacks Caranx ignobilis and Caranx melampygus (Carangidae) in Hawaii. Estuarine, Coastal and Shelf Science.

[114] Wetherbee BM, Holland KN, Meyer CG, Lowe CG (2004). Use of a marine reserve in Kaneohe Bay, Hawaii by the giant trevally, Caranx ignobilis. Fish Res.

[115] Uchida, R. N. & Uchiyama, J. H. *Fishery atlas of the Northwestern Hawaiian islands*. Vol. 38 (US Department of Commerce, National Oceanic and Atmospheric Administration, National Marine Fisheries Services, 1986).

[116] Papastamatiou YP, Itano DG, Dale JJ, Meyer CG, Holland KN (2011). Site fidelity and movements of sharks associated with ocean-farming cages in Hawaii. Mar Freshwater Res.

[117] Friedlander AM, Brown E, Monaco ME (2007). Defining reef fish habitat utilization patterns in Hawaii: comparisons between marine protected areas and areas open to fishing. Mar Ecol Prog Ser.

