# Supplementary material for: An Assessment of Mobile Predator Populations along Shallow and Mesophotic Depth Gradients in the Hawaiian Archipelago
Source: Sci Rep. 2017 Jun 20;7:3905. doi: 10.1038/s41598-017-03568-1 (PMC5478628; doi:10.1038/s41598-017-03568-1)
Supplement: Supplementary file 1 — Supplementary Materials [file 41598_2017_3568_MOESM1_ESM.docx]

**An Assessment of Mobile Predator Populations along Shallow and Mesophotic Depth Gradients in the Hawaiian Archipelago**

Jacob Asher^1,2,3^, Ivor D. Williams^2^, Euan Harvey^3^

# Supplementary materials

Table S.1: Canonical analysis of Principal Coordinates (CAP), leave-one-out Allocation of Observations to depth groups in the MHI and NWHI.

Figure S.1. Canonical analysis of Principal Coordinates (CAP) illustrating the structure of roving predators in the MHI (A) and (B) NWHI and depth. Analysis was based on zero-adjusted Bray-Curtis resemblance matrix calculated from square root transformed roving predator abundance (*MaxN*, hr^-1^). Open symbols represent MHI sites, closed symbols represent NWHI sites. Light grey triangles = shallow water (0 – 30 m), medium grey, inverted triangles = upper mesophotic (30 – 50 m), dark grey diamonds = lower mesophotic (53 – 100 m). Canonical analysis of Principal Coordinates (CAP) illustrating the structure of roving predators in the MHI (C) and (D) NWHI according to *a priori* depth and habitat categories. Right-hand panels display vector overlays of Pearson’s rank correlation of individual predator species with the CAP axes by region. Light back lines, lengths > 0.35. Dark black lines, lengths > 0.40. All plots generated in PRIMER 7.0.11 (<http://www.primer-e.com/>). Maps in the figure were generated using Adobe Illustrator CS5 (<https://www.adobe.com/products/illustrator.html>).

Table S.2. Pair-wise PERMANOVA comparisons for aggregate roving predator populations and select species. Monte Carlo

values presented when the number of permutations are < 50.

Figure S.2. Relationship between abundances (M*axN*, hr^-1^) of species identified in SIMPER analysis according to depth. The regression spline model for the 85^th^ percentile is shown. Spline model and plot outputs were generated in R statistical software, version 3.3.0 (<https://www.r-project.org/>, R Core Team, 2016). Solid circles indicate hard-bottom, open squares indicate unconsolidated sediment. Maps in the figure were generated using Adobe Illustrator CS5 (<https://www.adobe.com/products/illustrator.html>).

Figure S.3. A.) Bootstrap resampling, 50 bootstraps per group. Normalized environmental data, transformed into a Euclidean distance matrix Region (MHI, NWHI) x Depth Strata (shallow; upper and lower mesophotic), plotted mMDS. Shaded bootstrap regions, which represent measurements of centroid error: 95% confidence intervals, averages based on m = 4 dimensional metric MDS (rho = 0.994). B.) Principal Component Analysis (PCA) of normalized environmental variables plotted for the MHI and C.) NWHI. Individual samples representing sites binned into regional (MHI, NWHI) and depth groups (shallow water; upper and lower mesophotic). Correlations of habitat variables specified by vector direction and length. D.) Distance-based redundancy analyses (dbRDA) on roving predator assemblage abundances for the MHI and E.) NWHI. *Bubbles* are scaled to represent total predator relative abundances at each site. All plots generated in PRIMER 7.0.11 (<http://www.primer-e.com/>). Maps in the figure were generated using Adobe Illustrator CS5 (<https://www.adobe.com/products/illustrator.html>).

Environmental habitat variables were similar between the MHI and NWHI (Figure S.4A), as evidenced by the overlap of 95% confidence interval ellipses between the shallow water and upper mesophotic zones between regions. However, variable separation in the lower mesophotic zones was attributed (in part) to asymmetric sampling of hard-bottom versus unconsolidated sediment sites substrate types (i.e. disproportionate number of sand flat sites surveyed in the MHI), coupled with changes to biotic cover (e.g. diminished coral cover) and declining habitat complexity with depth.

C

The Principal Component Analyses (PCA), which assesses covariance along benthic functional groups for all pooled survey sites across the MHI (Figure S.4B) and NWHI (Figure S.4C), explained over 63.5% and 58.7% of the variation along the first two principal components respectively. Coral cover, habitat complexity, and turf algae were aligned along the first principal axis and tended to be higher in shallow water coinciding with shifts from aggregate reef, spur-and-groove, and boulder habitats to lower lying aggregate and patch reefs, rubble flats, or sand flats as depth increased. Macroalgae and crustose coralline algae cover were aligned with the second principal component and largely driven by previously described changes in sampled habitats when moving from shallow to mesophotic depths, along with shifts in increased unconsolidated sediment percent cover.

While 89.8% (MHI; Figure S.4D) and 86.4% (NWHI, Figure S.4E) of the fitted DistLM-dbRDA models were explained along the first two axes, only 10.5% and 18.6% of the total variation could be explained within each respective region. In the MHI, habitat complexity, depth, and % turf algae were identified as the main environmental contributory variables by the relationships between dbRDA coordinate axes and orthonormal X variables and three species (*Caranx melampygus*, *Carcharhinus plumbeus*, and *Seriola sp.;* Figure S.3D) being weakly correlated (Pearson correlation > 0.25), with assemblage vectors indicative of strength and direction. In particular *Caranx melampygus* was aligned with areas of increased habitat complexity, while *Seriola sp*. and *Carcharhinus plumbeus* were unsurprisingly correlated with increasing depth.

In the NWHI, % hard coral, % macroalgae, habitat complexity, and depth acted as principal, contributory variables. Seven species were correlated in patterns largely as previously described, with alignments noted for *Caranx ignobilis* and *Carcharhinus galapagensis* in shallower water in more complex environments, and the influence of depth on increased numbers of *Seriola sp*. and *Carcharhinus plumbeus* in mesophotic depths.
